# An Integrative Systems Perspective on Plant Phosphate Research

**DOI:** 10.3390/genes10020139

**Published:** 2019-02-13

**Authors:** Ishan Ajmera, T. Charlie Hodgman, Chungui Lu

**Affiliations:** 1 School of Biosciences, University of Nottingham, Sutton Bonington Campus, Sutton Bonington, Loughborough LE12 5RD, UK; ishan.ajmera@nottingham.ac.uk; 2 School of Animal, Rural and Environmental Sciences, Nottingham Trent University, Nottingham NG25 0QF, UK

**Keywords:** phosphorus use efficiency, regulation of phosphate uptake, systems biology, mathematical modelling, global food security

## Abstract

The case for improving crop phosphorus-use-efficiency is widely recognized. Although much is known about the molecular and regulatory mechanisms, improvements have been hampered by the extreme complexity of phosphorus (P) dynamics, which involves soil chemistry; plant-soil interactions; uptake, transport, utilization and remobilization within plants; and agricultural practices. The urgency and direction of phosphate research is also dependent upon the finite sources of P, availability of stocks to farmers and reducing environmental hazards. This work introduces integrative systems approaches as a way to represent and understand this complexity, so that meaningful links can be established between genotype, environment, crop traits and yield. It aims to provide a large set of pointers to potential genes and research practice, with a view to encouraging members of the plant-phosphate research community to adopt such approaches so that, together, we can aid efforts in global food security.

## 1. Introduction

Based on current trends, global agricultural production needs to double by 2050 to feed the projected increase in the human population [1]. With the advent of the Green Revolution, crop production in the past four decades has kept pace with the food demand [2], but this has been through unsustainable farming practices, including deforestation, increasing use of chemical fertilizers, biocides, and modern irrigation techniques. This has led to a loss of biodiversity, degradation of land quality, scarcity of fresh water and environmental pollution [3]. Consequently, in recent years there has been a stagnation or slowing of crop-yield growth [4]. Indeed, the challenge now is to revive the rate of agricultural production with minimal environmental impacts, limited resources and socio-economic constraints.

Currently, agriculture practice has become highly dependent on the use of fertilizers, without which global food production would reduce to half [5]. Particularly, the dependency on phosphate fertilizers has become unsustainable, as the stocks of phosphate ore are finite and are forecasted either to run out or to become prohibitively expensive in the next 5–10 decades [6,7,8]. Given the fact that phosphorus is a non-replaceable limiting resource essential for food production, global phosphorus security has a direct implication for global food security. In addition, the concern over sustainability of phosphorus also arises from the environmental issues including eutrophication caused by the runoff from fertilized fields [9]. Various possible approaches have been suggested and are being assessed to address this global challenge [10]. Most of these aim to improve fertilizer management strategies, develop efficient recycling processes or modify plant traits [11,12]. The latter has received greater attention for several decades, but the success in terms of practical application has been limited to date [13].

Given the above, this review begins with a short commentary on global perspectives, illustrates the extreme complexity of responses associated with phosphorus deficiency, the strategies for improving phosphorus use efficiency (PUE), and the need for translational research. Furthermore, this work introduces integrative systems approaches with particular emphasis on their prior applications and potential role toward informing the development of high-yielding phosphate efficient crop varieties.

## 2. Global Perspectives

In many parts of the world, changes to agricultural practice, namely, less but targeted fertilizer input, can sustain if not increase yields, and reduce both pollution and global warming effects. However, population growth is outstripping increased crop production in Northern and Sub-Saharan Africa and Western Asia, which may be a factor causing the migration of people from these regions to Europe. These areas require increased phosphorus input into the soil, but are hampered by economic and political factors, which are beyond the scope of this work. A recent position paper [14] goes into considerable detail about these issues. The situation can start to be improved with increased dialogue between global agronomists and both policy makers and funding agencies to ensure that biological research targets the aspects of most urgent need (i.e., for Africa and Western Asia). This can be thought of as translation from the field back to the lab. There is also a case for improving PUE in crops in the long term, so that better use is made of current soil phosphate levels and growth in poorer soils becomes a viable proposition. Given that it takes over two decades for laboratory discoveries to translate into government-approved commercial crop growth, beneficial research discoveries are needed urgently. Further information on this can be found in Supplementary Information.

## 3. Response to Phosphate Starvation

Phosphorus is an essential element in nucleic acids, phospholipids, phospho-proteins and metabolites. These encompass all physiological aspects of plant growth and development [15,16]. Unlike other macronutrients in the soil, the concentration of soluble mostly inorganic phosphate (Pi) is often low. This is due to its complex physico-chemistry, making it a major limiting factor for plant growth [17]. Owing to its low availability and slow diffusion in soil, the concentration of phosphate in soil solution remains quite low [18]. As a result, plants are often prone to phosphate stress and are endowed with counter mechanisms for their survival.

Being essential for growth and development, Pi uptake and utilization need to be tightly regulated. The concentration of cytosolic phosphate is generally thought to remain constant under normal circumstances [19], though on short time scales fluctuations have been observed [20]. This involves transport of phosphate between various inter- and intra- cellular phosphate pools, mainly via membrane-bound transporter proteins. When Pi is abundant, its rate of absorption exceeds demand. Under such conditions, most plant varieties prevent phosphate toxicity by reducing uptake from the soil, increasing Pi efflux and storage in the vacuoles [17]. However, some species (e.g., *Hakea prostrata*, blue lupin, and subterranean clover) have a very low capacity to downregulate their Pi-uptake system, showing toxicity symptoms under typical or high Pi supply [21].

Conversely, under phosphate limiting conditions, plants maintain cytosolic phosphate levels in several ways: facilitating the availability of external Pi, increasing its uptake, recycling and consumption of non-essential molecules containing phosphorus [20]. For example, secreted OsPAP21b from rice roots hydrolyses the bound organic phosphate sources and thus increases the availability of soluble Pi [22]. These processes principally take place at three sites, i.e., shoot, root and rhizosphere, but the precise sequence in which they act and integrate is still ambiguous. In the case of the shoot and root, these responses occur at different biological scales, i.e., morphological, anatomical, physiological and biochemical.

### 3.1. Shoot

With the depletion of vacuolar phosphate reserves, a lack of cytosolic Pi reduces photosynthesis [23], eventually inhibiting plant growth and development. Typical phenotypic symptoms of phosphate deficiency include stunted shoot growth and branching, dark to blue green coloration of leaves, weaker and thin stems, reduced tillering, imperfect pollination, fewer flowers, delayed maturity, poor grain quality and low yield [24]. Phosphorus deficiency in leaves may interfere with the normal opening of the stomata and compartmentation of Pi, leading it to being primarily in the cytosol and chloroplasts, presumably for metabolic processes [20,25]. It triggers senescence of older leaves and mobilization of Pi to younger leaves, meristems, flowers and seeds [26]. Moreover, starved plants translocate roughly half of the phloem-derived Pi back to the xylem [27].

At the cellular level, various physiological changes are triggered, such as reduced photosynthesis, increased sugar concentration, accumulation of anthocyanin, transfer of di-galactosyl-diacyl-glycerol (DGDG) from chloroplasts to mitochondria and release of vacuolar phosphate that may be insufficient to compensate for the decreasing cytosolic Pi levels [28,29]. Besides these, alteration in the expression of developmental and shoot-specific genes has been observed [30]. For example, the elevated expression of the *OsHAD1* gene in shoots has been shown to increase the phosphatase and phytase activity in response to low P in rice leaves [31]. However, the molecular mechanism underlying local phosphate sensing and signalling in the shoot remains unknown.

### 3.2. Root

Different plant species have evolved divergent adaptations to root morphology and exudation in response to Pi deficiency [32]. Persisting low P availability alters the Root System Architecture (RSA) by stimulating lateral-root development, causing an increase in specific root length, expanding the absorptive root-surface area by increasing both root-hair length and density, and, in some species, developing cluster roots and attenuating primary root elongation [32,33,34]. To add to the complexity, different cultivars of the same species show differing RSA responses to P stress, for example, subterranean clover [35].

Generally, RSA is under the regulation of developmental and hormone-related genes [36]. Cell division is perceived to govern phosphate demand in growing organs and determines the magnitude of expression of Phosphate Starvation Induced (PSI) genes [37]. On sensing low phosphate, a reduced rate of root cell elongation and progressive exhaustion of root meristematic cells cause attenuation of primary root growth in *Arabidopsis* [38]. Owing to the exhaustion of the primary root meristem, mitotic activity is shifted to the site of lateral root formation, thereby increasing their number [39]. Each lateral root then behaves like a primary root, eventually growing more lateral roots of its own [40]. The proliferation of lateral roots leads to shallow root systems allowing better exploration for Pi in the top soil [41]. Recently, it has been found that the rice *RMD1* gene controls crown-root angle under low Pi conditions in soil. The expression of *RMD1* is observed to increase in response to low Pi, which results in a shallower root system, hence enhancing Pi-foraging capacity [42].

Root-hair proliferation is arguably the most characteristic local response to phosphate deficiency, and it is regulated by an array of cellular and genetic processes [43,44]. Under phosphorus stress, the emergence of root hairs closer to root tips increases the root surface area, elevating the potential for Pi uptake [45]. In *Arabidopsis* and rice, root-hair elongation has been observed to be a low-phosphate adaptive-response regulated by auxin [46,47]. The final length of root hairs is suggested to be related to the level of respiration and metabolic activity in these cells, which is elevated under phosphate stress [32,48]. These cells may eventually die off, providing anchorage to the roots and use of their nutrients elsewhere in the plant. Along with root hairs, certain species in families, including Casuarinaceae, Fabaceae, Myricaceae and Proteaceae, form cluster (or proteoid) roots [49]. Internal phosphate is known to regulate cluster/secondary root formation [32]. Enhanced Pi uptake inhibits the formation of cluster/secondary roots, thereby removing the need to invest energy and material in their growth.

All the above changes are the result of various cellular and sub-cellular modifications. Thus, it is important to understand the fate of individual tissues in response to phosphate stress, especially the epidermis, pericycle and cortex, which respectively produce more and longer root hairs, more lateral roots and aerenchyma, whose Pi is utilised elsewhere in the plant [40,50,51]. Cell division and their rate of elongation are reduced, which significantly modifies the root anatomy, as observed in longitudinal and transverse sections from *Arabidopsis* [45]. Many of these architectural and anatomical adaptations have underlying molecular mechanisms which still remain ambiguous [29].

### 3.3. Rhizosphere

Plants also respond to phosphate deficiency by altering the biochemical environment of the rhizosphere [52]. This involves exudation of organic anions (malate, citrate and oxalate), enzymes (phosphatase, phytases), phenolic acids, protons and other molecules [33]. In general, these exudates mainly promote solubilisation of insoluble phosphate compounds, by competitively binding with the cationic phosphate partners and liberating the Pi ions from organic compounds [53,54]. Some exudates also promote recruitment of soil microbes by providing a carbon source [55], and/or acting as a chemo-attractants [56]. These microbes either trap Pi for the plants or release exudates in turn solubilizing organic and inorganic phosphate compounds [33]. With few exceptions, including *Arabidopsis*, many plants species use fungal symbionts, Arbuscular Mycorrhizal Fungi (AMF), to enhance foraging and acquisition of Pi and other nutrients. AMF grow within root cortical cells and extend hyphae far into the soil, eventually leading to the inter-plant root-hyphal network [57]. Influx of Pi in roots colonized by mycorrhizal fungi is 3–5 times higher than in non-mycorrhizal roots.

In response to low phosphate, exuded strigolactones, in both *Lotus japonicus* and rice, enhance hyphal branching and root colonization of AMF, consequently increasing the exploration for Pi [58,59]. Upon AMF colonization, some Pi transporters are repressed, particularly in the epidermis, while several phosphate-starvation-induced genes are activated, including P-type H+ ATPase, mycorrhizal-induced Pi transporters and phosphatases [29]. Furthermore, with the high availability of Pi in the soil, the rate of AMF colonization decreases, potentially due to the increase in internal phosphate level [57]. The benefits of AMF were significantly less pronounced in plants with longer root hairs [60], perhaps because the latter also increase cytosolic Pi. Besides Pi uptake, the AMF also influence root system architecture, most prominently, by enhancing lateral root formation [61]. The mechanism of AMF colonization and its associated effects on Pi uptake and RSA have been extensively reviewed [62,63]. Further information on phosphorus dynamics in soil is provided in supplementary information.

## 4. Spatio-Temporal Interaction between Phosphate Starvation Responses (PSRs)

**Figure 1:**
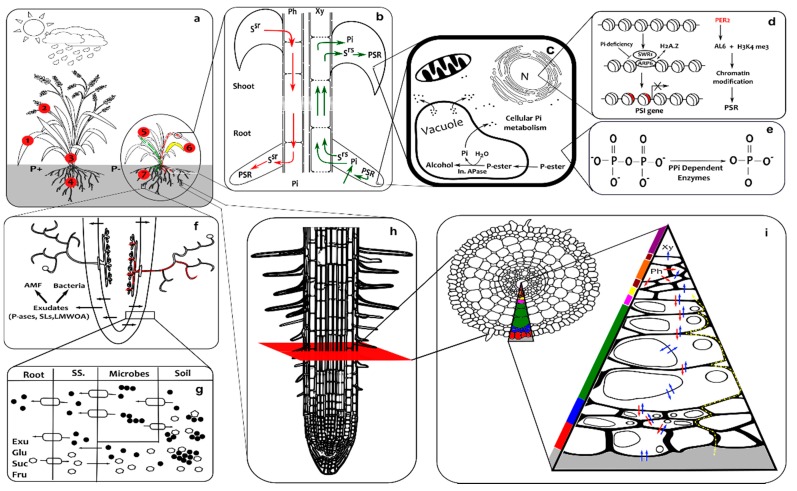
Integrated overview of phosphate starvation responses. The responses and signalling mechanisms operate at a range of scales and different locations which are depicted in nine connected panels: (**a**) denotes the whole plant and field scale; the numbers in red circles represent normal and low inorganic phosphate conditions (1) phosphorus playing a major role in various plant growth and developmental process including photosynthesis; (2) the highest level of Pi being found in the vegetative parts of the young plant, which upon maturation, moves into (3) fruit and seeds; (4) root development correlating with Pi levels; (5) Pi helping to increase water use efficiency and disease resistance; (6) abnormal leaf discolouration under low Pi conditions; and (7) shallow root system with more root hairs and lateral roots. (**b**) denotes the whole plant scale with systemic signals Systemic Shoot to Root (SSR) from shoot through the phloem to the root and Systemic Root to Shoot SRS) from the root to the shoot through the xylem; Pi, and water and other nutrients also go up to the shoot by this route. (**c**) denotes cells from any part of the plant which respond to phosphate deprivation altering the lipid content, releasing phosphate stores from the vacuole where Pi is liberated from esters by Acid Phosphatases (APase). (**d**) denotes the epigenetic effects (principally chromatin modification) that influence transcription of Phosphate Starvation Response genes. (**e**) denotes the pyrophosphate-dependent glycolytic bypass enzymes and metabolic Pi recycling system. (**f**) denotes rhizosphere activities, specifically the exudation of acid phosphatases (P-ases), Strigalactones (SLs) and Low Molecular Weight Organic Acids (LMWOA) which stimulate bacterial activity and attract Arbuscular Mycorrhizal Fungi (AMF) that form arbuscular structures within the root – mycorrhizal delivery of Pi is depicted in red. (**g**) denotes a close-up view of the rhizosphere boundaries between the root, soil sheath (SS), microbes and soil where exudates and sugars (Glu–glucose, Suc–sucrose and Fru–fructose) are secreted through efflux transporters respectively to solubilise Pi compounds and stimulate bacteria to do the same, l and Pi is imported through transporters of varying affinity; the exudates/sugars, transporters and Pi are respectively depicted by hollow ellipses, lozenges with directional arrows and black circles. (**h**) denotes the alteration in meristem and elongation zone length and the formation of root hairs. (**i**) denotes a cross section through a root and the paths taken during Pi uptake: the positions of different tissues within a root, namely, epidermis, exodermis, schlerenchyma plus cortex, endodermis, pericycle, phloem, cambium and xylem are marked respectively by red, blue, green, pink, yellow, orange, pale brown and purple; and transport of shoot-to-root signal molecules, symplastic/inter-organellar Pi and apoplastic Pi are depicted respectively by red, blue and dashed yellow arrows.

**Figure 2:**
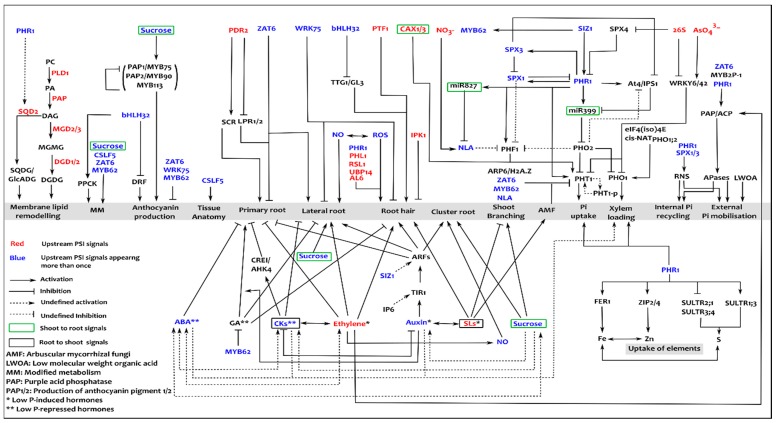
Molecular signalling in phosphate starvation responses. The different responses are specified in the grey bands, with hormonal regulation and transcription factor/signal/enzyme regulation delineated respectively below and above; the upstream signal and regulators are denoted in red and blue depending on whether or not they feature one or more times in the network, with some in green and purple boxes respectively to denote shoot-to-root and root-to-shoot systemic signals; the types of edges (interactions) and meaning of the acronyms are shown in the key on the lower left.

All the above Phosphate Starvation Responses (PSRs) act at different temporal and spatio-physical scales, i.e., field, rhizosphere, plant, organ, tissue, cell and sub-cell (Figure 1). Plants integrate intrinsic and extrinsic factors, eliciting such responses to counter phosphate stress. This relies on both local and systemic sensing/signaling mechanisms that monitor external and internal phosphate status. External Pi is sensed by a local system around the root-tip [64], particularly in the root cap [65]. This independently attenuates primary-root growth and promotes root-hair development in *Arabidopsis* [66]. Along with genetic regulation, modulation in the dynamics of different hormones plays an important role in such local responses, leading to altered RSA, Figure 2.

Internal phosphate status is governed by systemic signalling, to increase Pi availability, recycling, uptake and transport [67]. This involves metabolic reprogramming, degradation of expendable nucleic acids and de-repression of high-affinity Pi uptake and xylem-loading transporters [68], Figure 2. Moreover, lateral and cluster root growth is also partially regulated at a systemic level [29]. Systemic signalling (Figure 1b) integrates the local responses across the plant by trafficking various signals through the vasculature. This encompasses phloem-mediated shoot-to-root signals (microRNAs, sugars and CAX-Ca^2+^/H^+^ transporters) and xylem-mediated root-to-shoot signals (Pi, cytokinins and strigolactones). These signals collectively trigger a cascade of responses involving a large number of PSI genes [67,69]. Most of these are depicted in Figure 2 and have been elaborately reviewed [29].

PSI genes are classified as early or late in expression (i.e., within a few hours or after one day of Pi depletion), and whether they are shoot-, root-or non-specific. In *Arabidopsis*, the early genes encode transcription factors belonging to MYB, ERF, WRKY and bHLH families, Pi transporters, protein kinases and proteins/enzymes initiating exudation, membrane remodelling and lateral root formation (whose emergence is not until later times). The late-responsive genes mainly encode the downstream regulators for Pi transport, recycling and metabolic bypass processes [66,70]. In roots, persisting low Pi elicits genes involved in Pi uptake, exudate synthesis and importantly, hormone regulation leading to altered RSA. In addition to PSR genes, chromatin remodelling, post-transcriptional and post-translational modifications also play an important role in regulating PSRs.

Most PSRs aim, at least in part, to increase Pi uptake and transport in the plant. Furthermore, the tissue-specificity and phosphate-responsive expression of these genes, as presented in Table 1, reveal a greater level of complexity in the system. The core pathway underlying transcriptional regulation of Pi acquisition involves the dissociation and sequential sumoylated activation of a Phosphate-Responsive transcription factor, named PHR1 in *Arabidopsis*, and its orthologue PHR2 in rice [71,72,73,74]. This triggers a network of molecular responses, including gene activation, microRNA-mediated repression, a reduction in directed ubiquitination and active trafficking of Pi transporters to the plasma membrane [75]. PHR1/2 and its associated pathways have been extensively studied (Figure 2) and reviewed [29,76,77]. Besides these, molecular processes such as chromatin remodelling, post-transcriptional and post-translational modifications also play an important role in regulating PSRs [78,79,80]. 

The transcriptional regulation of PSRs appears to be largely conserved between rice and *Arabidopsis* [81]. However, widespread and mitotically heritable changes in DNA methylation have been identified in rice, in contrast to the very limited changes in *Arabidopsis* in response to Pi stress [80], thus signifying the divergence in the mechanism regulating PSRs between these two species. After altering gene expression, phosphate stress elicits changes in chromatin patterns, almost entirely targeting transposable elements surrounding the genes [80]. Resetting was also observed in response to Pi deprivation in *Arabidopsis*. Following 21 days of starvation, the expression of 40% and 80% of induced genes was reversed within 1 and 3 days of resupplying of Pi, respectively. However, a few genes remained differentially regulated even after 31 days of recovery. In addition, reversion of chromatin states has also been observed upon Pi starvation recovery. Interestingly, the expression of genes encoding DNA methyltransferases in *Arabidopsis* is directly controlled by the key regulator PHR1 [82].

Phosphate deprivation triggers various metabolic modifications, especially in the shoot, to increase its mobilisation and reduce its utilisation [83,84], primarily by affecting photosynthesis, glycolysis and respiration. Such alterations in metabolism result in a lower requirement for Pi or adenylates, conversion of phosphorylated glycolytic intermediates to sugars and organic acids and modulation of various primary and secondary metabolite concentrations [85,86]. The primary metabolites include polyols, organic acids, amino acids, polyhydroxy-acids, fatty acids, nitrogenous compounds and organic phosphates, while secondary metabolites include glucosinolates, benzenoides, phenylpropanoids and flavonoids [87]. Offering a link between genetic and metabolic regulation, a recent study in *Arabidopsis* has suggested PHR1 is a prominent factor for metabolic reprogramming under phosphate stress [87]. However, the molecular mechanism underlying this interplay is yet to be deciphered.

Interestingly, the concentration of sugars (particularly, sucrose) increases in the shoot in response to low Pi [89]. This has implications for the transcriptional reduction of photosynthesis [90] and transcription-mediated elevation of sulfolipids, galactolipids, phosphatase, RNase, phosphoenolpyruvate carboxylase (PEPcase), anthocyanins and Pi transporters [91,92]. Such alterations recycle substantial amounts of Pi by compensating for phosphate precursors, protecting nucleic acids and chloroplast against photo-inhibition and facilitating Pi availability in the rhizosphere [25,93]. Increased shoot sucrose up-regulates the expression SUC2 transporters, which transfer sucrose to the phloem [94] serving as a systemic signal [95]. This correlates with the expression of various PSI genes underlying several root and rhizosphere responses described above [30,96,97,98].

In recent times, the elemental composition, the ionome, of tissues has been identified as a multivariate signature defining the specific physiological state of the plant, including phosphate stress [99,100,101,102]. The correlated accumulation of different elements is largely driven to maintain vacuolar and cytoplasmic osmolarity and charge balance, and also serves as a detoxification mechanism [103]. In *Arabidopsis* leaves, Pi limitation has been observed to increase the concentrations of B, Zn and As and decrease the concentration of P, Cu and Co [104] while in maize leaves, it significantly increased the concentration of K, Mn, Zn, V, Ni and Rb and deceased the concentration of S, Mg, Ca, Mo, Sr, Li and As [105].

Different cell types accumulate certain elements in varying amounts in their vacuoles. The role and mechanisms underlying the cell-specific distribution of different elements in plants are poorly understood. Although the location of element accumulation is fairly robust, alterations in expression of certain solute transporters, through genetic modification or by growth under stress, result in perturbations to these patterns [103]. For example, it has been shown that Pi limitation increases arsenic uptake via high affinity Pi transporters, while N deficiency increases Pi uptake via a miRNA/NLA signalling pathway. More such examples have been reported [106,107,108]. Furthermore, the large natural variation in the genetics of the plant to handle the combination of stresses [109] makes it difficult to investigate the crosstalk between Pi and other stresses.

It has recently become clear how cellular Pi levels are sensed. Two recent studies have reported that inositol polyphosphate signalling molecules (InsPs) act as the sensor. The binding of InsPs to proteins containing SPX domains enables them to interact with their target proteins, which are involved in regulating Pi uptake, transport, and storage [110,111].

## 5. Current Strategies and Challenges Towards Improving PUE in Plants

At the field scale, incremental improvements in PUE have been achieved through improved soil management [112], cultivar screening [35] and selective breeding based on improved root systems [113,114,115]. The potential routes to improve Phosphate-Acquisition Efficiency (PAE) include modification of RSA, root anatomy, rhizodeposition, rhizosphere-microbial interaction and Pi uptake. Phosphate-Utilisation Efficiency (PUtE) involves optimisation of harvest index while reducing plant phosphorus demand and/or enhancing its internal utilisation/recycling. PAE and PUtE combine to give an overall Phosphorus-Use Efficiency (PUE) for a plant. Far more progress has been achieved toward understanding the mechanisms underlying PAE than PUtE, perhaps because of the greater complexity of the processes involved. Various target genes or traits for improving PAE and PUtE have been tested [29,116,117,118,119], and the costs and benefits of different P-uptake mechanisms [120] have already been reviewed.

Transgenic approaches have been successful in introducing single genes to improve mostly PAE, at least, offering a proof-of-concept for their utility [121]. On various occasions, the results have not been reproducible or have negatively affected other traits. Hence, it is currently unclear how to predict the trade-offs. Genome-Wide Association Studies (GWAS) and especially determination of Quantitative Trait Loci (QTLs) have linked root traits with PAE in rice, wheat, common bean, *Arabidopsis*, soybean, barley and maize [122], and some have become the focus of breeding programmes [123,124]. In addition, a protein kinase gene, *PSTOL1*, has been identified in a low-P tolerance QTL, Pup1, in rice. This gene enhanced early root growth, enabling more uptake when incorporated into Pi-sensitive varieties [125,126]. Altogether, the low success rate of genetic manipulation for developing phosphate-efficient crop varieties [127,128,129,130,131] presents the need for smarter approaches.

## 6. Need for Integrative Systems Approaches

A systems approach views processes, behaviours and phenotypes as a mechanism (termed a system) in which both its components (nodes) and their interactions (edges) are defined. Where these interactions result in feedback circuits, the system becomes complex in the mathematical (and often the common) meaning of the term and can lead to counter-intuitive behaviour. For example both knockout and over-expression of a gene might exhibit the same phenotype [132]. In biology, a node can represent a type of molecule, pathway, cell, tissue, organism and population, depending on the physical scale of the system of interest. From this, it is clear that a node at one scale may form a system when considered at a lower scale. Likewise, above the biological (plant) population, there are local (field), national and global scales.

Put simply, integrative systems approaches are activities that address an issue by considering it as a system and employing multi-disciplinary expertise toward its study. The development and analysis of models are characteristic features of systems approaches. In this context, a model is a simplified representation of a dataset or a system, which provides a quantitative understanding of the data or system. A ‘data model’ reveals structure and relationships within a dataset. Biological examples include QTL analysis and GWAS, but also inferred networks derived from omics data [133,134,135].

A ‘system model’ represents the mechanism of the system and can either be static or dynamic. The former is simply the interaction network, while the latter quantitatively represents the combined rates of change of its components as a result of their interactions and system inputs and can explain unexpected behaviour. These models can also be extended to include the physical structure of the system in two or more dimensions, allowing even organ and whole plant growth to be modelled. All these modelling approaches have already been extensively reviewed [136,137,138,139].

There are four main reasons for developing these models [139]. The first is to test current understanding to see if it stands up to quantitative scrutiny. Often this shows that there is a gap in current knowledge, suggesting areas for further laboratory and or field study. The second is that they provide a platform to carry out in silico experiments to predict behaviour under many more circumstances than would be financially viable in-vivo. Hence, the third reason is to find out what are the most incisive experiments to carry out to make useful discoveries. The fourth reason concerns occasions when laboratory experiments are difficult or impossible, e.g., in establishing mycorrhizal systems. In such instances, modelling provides a mechanism to infer what might happen under various circumstances.

## 7. Current Systems Activities in Plant P Research

Following the pioneering work by de Wit in 1959, there has been a constant effort to develop and improve crop models that predict the performance of the genotype and assess the design of the adaptive strategies for given environmental conditions [140]. A large number of mathematical and computational models have been developed, particularly over the last 10–15 years, improving understanding of various aspects of plant processes at all scales of biological organisation [139]. With regard to resource acquisition by plants, a range of models at various physical (generally, supra-tissue) scales has been developed. These represent aspects of uptake of nutrient, water or contaminants [141,142,143,144,145,146,147,148,149,150], and the effect on soil nutrient availability of microbial [151,152,153] and exudate dynamics [154].

Pertaining to phosphorus, the current crop models include the concept of PUE, but only in terms of movement of phosphorus from one part of the plant to another and ultimately into seeds. However, there is no connection made to the genotype of the plant. Most plant-scale models have focused on phosphate dynamics in soil, to identify ways of optimising its availability [155,156,157,158,159]. In the context of Figure 2, this includes models representative only of panels a, f and g. Such models have highlighted the fact that when soil factors determine the availability of Pi at the root surface, the effect of having more transporters becomes negligible. The number and type of transporter are crucial, but primarily for avoiding Pi toxicity. Furthermore, modelling has shown that a small optimisation of RSA can lead to a large increase in Pi acquisition [160,161], PAE can be enhanced by increased root-hair length and longevity rather than their density [120] and root cortical aerenchyma tissue is beneficial for PUE by reducing metabolic and exploration cost [162].

A key recent paper has applied systems approaches to study the regulation of Pi uptake at the molecular and “whole root” scales [142], incorporating aspects of Figure 2 panels b, d and e. The interplay between laboratory and modelling work revealed knowledge gaps regarding the kinetics of components and the prediction of three new regulatory features: a Pi-mediated RNA-stabilisation mechanism for a regulatory long noncoding RNA, autoregulation of the ubiquitin-ligase gene (*PHO2*) and a Pi-sensitive co-regulator of this same gene.

Traditionally, forward genetics was used to identify genes by cross-breeding and phenotypic screening, which are very time consuming. Omics technologies are a form of integrative approach, as they bring together data notionally for all genes, transcripts, etc. Computational techniques have allowed these datasets to be brought together (in databases) for comparison and advanced data modelling (to produce interaction and inferred regulatory networks). These activities allow prioritisation of large gene lists so that trait-related genes can be found in much shorter timescales [134,163]. Perhaps the most advanced integrative tool at present is TraitCapture [164], which represents growth phenotypes/traits using Functional Structure Plant Models and links them with QTL data.

## 8. Next Steps

Improving PUE is essential to reduce environmental impacts, increase the nutritional value of grains and improve farm economies. However, current breeding strategies have had little success, owing to a poor understanding of the molecular mechanisms underpinning traits and their interactions [165]. Integrative systems approaches can assist in this area by helping to identify the components of (and their relative contributions to) traits of interest. Figure 1 depicts the phosphate-related systems from molecular to field scales and provide a starting point for integrative research into improving PUE.

From the outset, plant-phosphate biologists should be working closely with mathematicians and computer scientists to define and report quantitative data such as growth conditions (light regimes, growth matrices, etc.), intra- and extra-cellular concentrations of relevant metabolites/ions, and when known, the corresponding binding and/or kinetics parameters. The regulatory feedback response model [142], through its PHR1/2 variable, can readily be used as the basis for studies that link Pi uptake to external Pi mobilisation, root-hair growth, internal Pi recycling, membrane-lipid remodelling and the uptake of other nutrients. Adding MYB72 (regulated by the SIZ1 variable) allows the model to integrate uptake with modified metabolism, anthocyanin production and mycorrhizal interactions. SIZ1 itself is implicated in a host of other stress responses, paving the way for modelling the interactions in multi-stress responses. These molecular scale models can also be embedded in a multicellular/multiscale model, using software environments such as OpenAlea [166], VirtualLeaf [167] and Framework Models [168].

New types of experiments need to be designed (in collaboration with modellers and statisticians) that aim to capture the spatio-temporal and quantitative characteristics of relevant processes and their surrounding subsystems, i.e., time-series data for multiple cells/tissues, so that rates and quantities can be determined. A comparison of these processes in related genotypes (particularly, those performing well in low-P landscapes) or multiple conditions, for example, a range of constant external Pi levels, will also be both informative and closer to field conditions. The use of novel drugs (e.g., Phostin and Phosphatin) and Pi analogues (e.g., phosphite and methylphosphonate) could be instrumental in further deciphering plant responses to Pi starvation [20,65,169,170]. With respect to the phosphate-starvation responses, much of the core gene regulatory network involving PHR1/2 has been identified in *Arabidopsis*, and these genes appear to be conserved across wide range of plant species [171,172]. Thus, Table 2 provides a set of candidate genes/molecular components for crop improvement, offering a starting point for integrative research for improving PUE.

At the molecular scale, omics techniques help to identify the components of the system concerned but note that individual omics techniques can give a misleading impression of which players are important. This can be resolved by using multiple omics techniques on the same samples [176]. This will help to refine and prioritise the regulatory pathways shown in Figure 2 and provide the interaction topology on which dynamic models can be developed. The initial dynamic models should focus on smaller parts of the overall network shown in Figure 2, as outlined above. By careful design from the outset, especially using standard terms for the variables, these models can be integrated later, so that how the different aspects of the PSR affect each other can be discovered.

Suitable models to predict improvements in PUE must span the physical scales shown in Figure 1. The modelling should start by linking adjacent scales before integrating across them all. Potentially, multi-scale models could be used to assess the long-term impacts of a genotype on soil nutrient dynamics, crop productivity and sustainability of the cropping system for a wide range of environmental conditions. However, multiscale models require multiscale data for both model development and testing of predictions. A detailed illustration on the generation of multi-scale data (from a whole-plant to the sub-cellular scale) and development of corresponding multi-scale models can be found in the review by [177].

Vacuoles are the main phosphate store and provide a buffer for the cytosol during variable Pi input [178]. Elevated storage could potentially improve PAE. Although the transporter proteins have been identified, only a little is known about their transcriptional or post-translational regulation, and a current weakness is the difficulty of differentiating cytosolic from vacuolar phosphate concentrations in vivo. A credible solution is the use of transgenic Pi nanosensors (for example, [179]). With time-series data, mathematical modelling can begin immediately to explore hypotheses for the regulation of vacuolar transporters and Pi flux, even on transient timescales. In vivo nanosensors of ATP can also play a role [180]; though under phosphate stress, cells can attempt to maintain ATP levels by using adenylate kinase to convert ADP to ATP + AMP. Hence, a nanosensor for the latter could be more useful.

In the long term, it is likely that a complete and explicit understanding of the molecular basis of PUE would still be insufficient. This is because the mechanisms for Pi uptake and utilisation affect other regulatory subsystems and vice versa. For example, the regulatory mechanism underlying Pi uptake links to salt tolerance and the uptake of other nutrients are well-established [181,182,183]. In view of this, identifying genotypes appropriate for different soils, environmental conditions and agricultural systems are needed. Therefore, it will also become necessary to study the effects of improving PUE on other traits. Finding environment-specific optima among these competing effects to achieve adequate crop yield is a daunting challenge. By integrating data and knowledge into models, systems approaches form the best way to find such optima and can be helpful in addressing the open questions pertaining to Pi sensing and signalling.

Under field conditions, plants are confronted with a combination of stresses [184], which elicit non-additive responses. The latter are often unpredictable and cannot be extrapolated from studies of individual stresses in the laboratory [128]. However, mimicking such environments in the laboratory and evaluating the importance of specific processes/traits are likely to be very difficult, but will provide a more realistic view of plant responses. Yet again, the laboratory and modelling work should begin by studying pairs of stresses, for example, Pi deficiency with drought or heat or nitrogen stress. The outputs of such experiments are very large multi-dimensional datasets, for which advanced computational techniques will probably be essential.

Along the lines of TraitCapture [164], advanced integrative pipelines could be developed that represent PUE phenotypes/traits using “Functional-Structural Plants Models” [185] and linking them with QTL data. This approach does not necessarily target specific genes nor reveal the mechanisms contributing to traits. However, it is a step in the right direction as genes/functions could be incorporated later. The bottleneck concerning high-throughput root phenotyping could be addressed using the advanced methods and platforms reviewed in [186,187].

## 9. Conclusions

The research to develop high-PUE crop varieties is hampered by extreme complexity on many fronts. The latter ranges from molecular interactions in both soil and plants, through agricultural practice, to national and geopolitical issues surrounding the cost of P supply. Notably, plant responses to low Pi may trigger other stresses and consequent adaptive responses. It is no surprise, therefore, that attempts to improve PUE have had little success because various spatio-temporal factors/traits and their interactions need to be taken into consideration.

Models (mathematical or computational) are able to represent and explain complex behaviour, meaning that systems approaches have a good track record of making important novel discoveries in comparatively short timescales. In the case of the crop models, they continue to have practical benefit to farmers. Clearly, integrative systems approaches should be brought to bear in phosphate research, so that the open questions remaining in this area can be addressed. Modelling can help in ranking potential target genes (and combinations thereof) on how likely they are to elicit the desired phenotype or trait. In addition, it can be used to explore different trait combinations and their interactions with the environment, leading to the choice of the most suitable ideotype for given field conditions.

Multidisciplinary groups working on the different aspects of phosphate research (particularly at the different scales) must come together and share terminologies, skills and concepts, so that models linking genotypes to desirable traits can emerge. Furthermore, practical benefits are likely to accrue faster if these projects turn away from purely scientific endeavours to include application or translation of results to crop species. Identifying the most appropriate projects is likely to come through increased engagement with industrial companies, farmers and governmental licencing agencies, i.e., linking to the scale above the phosphate-research community.

## Figures and Tables

**Table 1:** Expression of PSR genes in rice root under phosphate sufficient and deficient conditions.

Gene	MSU Id (LOC_Os)	P+			P-(Whole Root)
		EES	Cortex	EPS	
*ARP6*	01g16414				**→**
*bHLH32*	03g15440				**→**
*CAX1*	11g05070				**↘**
CAX3 **	02g21009				**↗**
*IPK1*	04g56580				**→**
IPS1 **	03g05334				**↗**
*LPR1*	01g03530				**↘**
MYB62 **	01g03720				**↗**
*PHF1*	07g09000				**↗**
*PHO1*	02g56510				**↗**
*PHO2*	05g48390				**↘**
*PHR1*	03g21240				**→**
*PHR2*	07g25710				**→**
PHT1;1 **	04g10800				**↗**
*PHT1;2*	03g05620				**↗**
*PHT1;4*	04g10750				**↗**
*PHT1;6*	08g45000				**↗**
*PHT1;8*	10g30790				**↗**
PHT2;1 **	02g38020				**→**
*PLD1*	05g29050				**→**
*PTF1*	06g09370				**→**
*SIZ1*	05g03430				**→**
*SPX1*	06g40120				**↗**
*SPX3*	10g25310				**↗**
*SQD1*	07g01030				**↗**
ZAT6 **	03g32230				**↗**

**Very high** **High** **Medium** **Low** **Very low**
**Nil**
EES: Epidermis, Exodermis, Sclerenchyma; EPS: Endodermis, Pericycle, Stele. ** Overall very low expression, relative expression between tissue types is shown. Directional arrows depict no change (**→**), upregulation (**↗**) and downregulation (**↘**), in the transcript levels following 21 days of phosphate stress (P−). These expression levels are relative to the phosphate sufficient condition (P+) at day 21 and are adopted from the published mSEQ dataset [81]. Note: The table is generated using the dataset available from the RiceXPro database [88]. This gene expression dataset represents different tissue types from root tip and elongation zone, under normal growth conditions.

**Table 2:** Phosphate Starvation-Induced genes in *Arabidopsis* and their respective orthologs in different crop varieties.

Gene	*Arabidopsis*	Rice	Maize	Sorghum	Brachy	Wheat	Barley	Soybean	Tomato	Potato	Brassica	Grape	Medicago	Populus
Prefix	At	LOC_Os	GRMZ2G	Sb	Bradi	Traes_	MLOC_	GLYMA	Solyc	PGSC0003 DMG4000	Bra0	VIT_	MTR_	POPTR_0
*** ARP6***	3g33520	01g16414	088487	08g21780	2g10130	2BL_221EC0BB1	64804	04G07540 06G26590	05g018600	14966	30818	11s0016g05490	ND	018s12840 002s0340
						1BL_51C489EEB								
						2AL_4DCD06BE9								
						2DL_E17A1BB12								
*** At4,*** ***IPS1***	5g03545	03g05334	086179	ND	ND	4AL_D7C83DB52	ND	ND	ND	ND	ND	ND	ND	ND
	3g09922	01g0838350 **	843352			4BL_00AAC9279								
*** bHLH32***	3g25710	03g15440 01g06640	043854 088443	01g040450 03g005250	1g67500 2g03830	4DL_7BD3165FB	66385	12G14400	07g053290	23577	26510 25158	10s0003g01160	5g066080	004s05480
						4AS_DD16F89E7								
						4BL_A2B32FCEE								
*** CAX1***	1g08960	11g05070	004414	08g002860	4g42870 4g42880	5BL_6A7BE3F0C	21103 16013	03G39920	01g098800	28607	31650	14s0128g00240	7g113730	013s02590
						5AL_97C4C3E20								
						5DL_B669983C4								
						5AL_B925FC757								
*** CAX3***	3g51860	01g37690 02g21009	011592	03g024820 04g010130	2g41090	2AL_26065F906	17450 13658	01G30610	09g005260	11787	12833	08s0007g02240	7g068380 4g016720	006s10080 016s12290
								01G30643						
						2BL_445F5B3B7		03G07910						
						2DL_2A2F4F62B		03G07950						
								18G43000						
						3AL_3CA77B366								
*** IPK1***	5g42810	04g56580	150496	06g31650	5g24890	2AL_55460AE54	38910	14G07880	04g080670	03698 15950	27458	18s0001g12910	ND	005s25180
						2BL_A465A5769								
						2DL_EFEF40224								
*** LPR1***	1g23010	01g03530 01g03549 01g03620	086727 054050	03g007480 03g007470 03g007440	2g01850	4DS_DE0D4FCF6	79739	20G26270 10G41010	05g008290	30551	24558	01s0011g04720	ND	010s12440
						4BS_ECDA94252								
						1BS_5144C54E4								
						4BS_06EE678FF								
*** miR399***	2g34208	* osa MIR399- a-k	* zma MIR399- a-j	* sbi MIR399- a-k	* bdi-MIR399 a-b	* tae-MIR399	* hvu-MIR399	*gma-MIR399 a-h	* sly- MIR399	*stu-MIR399 a-o	* bna-MiR399 a-c	* vvi-MIR399 a-i	*mtr- MIR399 a-t	* ptc-MI399 a-e
	1g63005													
	5g62162													
	2g34202													
	2g34204													
	2g34208													
*** MYB62***	1g68320	01g03720	096358 162709	03g007360	2g01960	3AS_0A8F6B069	65555	10G41930 20G25110	05g009230	14550	04041 04297	01s0011g03730	1g110460	008s12130 010s13290
						3AS_5E49D447AF								
						3B_5033C9A8								
*** PHF1***	3g52190	07g09000	158489	02g005080	1g55000	7BL_48A3E8A28	22417	10G34380	11g007780	* G404027324	06909	13s0074g00010	ND	008s02920
						5DL_42842D4B4		20G33180		* G402027324				010s23870
*** PHO1***	3g23430	02g56510	466545	04g036730	3g54920	6DL_C4D2B23A	12153 56639	10G00720	09g090360	17163	14948 23727	05s0049g01410	2g077690 1g075640	008s16930 010s07970 008s18760
						6BL_6D7561359		10G32670						
								20G34390						
						6BL_0325BEAA4		02G00640						
						6BL_CB0A10DC9								
*** PHO2***	2g33770	05g48390 05g48400	381709	09g028110	2g16960	1DL_F122253A3 1AL_E5224B6EA 5BL_196F297B6 1BL_241A3B9EF	53410	07G31360	02g078210	29724	21874	00s1927g00020 00s0265g00070	2g013650 4g020620	004s04350 011s05240
								13G24810						
								13G31290						
								15G08040						
***PHR1***	4g28610	03g21240 07g25710	006477 162409	01g036440 02g010520	1g63530 1g28920	3DS_0240F189D 4AS_7220D33B3 3AS_6EEB8D2B2 3B_6780F56DD	5585 60198	19G35080 10G04540 03G32350	06g008200 09g072830	23467	10355 11042 24188	07s0005g04120	7g098250	002s25960
*** PHT1;1***	5g43350	04g10800	170208	06g002560	5g02750	4AL_81AC819EF		02G0084010G00950	03g00530 03g00560	13403	27491	05s0049g00920 05s0049g00930	1g043200 1g043290 1g043220	0010s08270
								10g33030			35977			
								20G34616			33675			
*** PHT1;4***	2g38940	08g45000	045473 154090	06g002800 01g046900 01g020570	5g02730 3g27680	4AL_C56125840		03G31950	06g034200	03798 03839 10288 10289 17503	0107 5069 5070 5071 17093 17275	07s0005g03290 07s0005g03300	5g068140	005s24500 005s24510
		04g10690						10G04230						
		03g05610						13G18421	09g066410					
		10g30790						19G34710	09g073010					
*** PHT2;1***	3g26570	02g38020	092780	04g024630	3g47550	6AL_294A9853 2	6818	08G38840 18G20870	05g013510	18604	25212 34242	00s0291g00060	8g069390	008s19070
						6BL_6BC705098								
						6BL_5C168B1DD								
						7DL_53DB0C6B3								
*** PLD1***	3g16785	05g29050 01g20860	066485	01g017850 03g012720	2g27950	1AL_11AB1B224	70374	09G04620 15G16270	01g065720	09598	22178	05s0077g01830	1g083620	008s23240 010s00850
						1BL_884FA4347								
						1DL_AA198212F								
*** PTF1***	5g58010	06g09370	024530	10g006250	1g46700	7AS_D56F0F9E5	59503	06G38935	12g100140	22058	20398 06788	11s0016g00380	3g027650	006s13790
						7BS_13A10F94A								
						7DS_68E3ACF8E								
*** SPX1***	5g20150	06g40120	171423	04g006990 10g023590	1g36610 3g07470	7AL_2DSB863A7	54859	06G07260	12g009480	02890	06543 20088	11s0016g05330	1g012440	006s06880 018s13140
						7BL_DD88849AE								
						7DL_B994066FA								
*** SPX3***	2g45130	10g25310 03g29250	370780	01g032880 01g023270	1g60250	2BS_E9B276FE4	62152	01G33170 03G03820	01g090890	2601	00373 40324	05s0077g00270	ND	002s14470 014s06020 017s00240
						7AL_40678A3B5								
						2AS_96D993EDD								
						7DL_AC271F4EE								
*** SQD2***	5g01220	07g01030 01g04920 03g15840	100652 049190	02g000240 03g006480 01g040150	1g59860 2g02800 1g67200	3B_4DEB64E2E	63045 12521	07G17680 01G27840 03G14200	09g014300 10g085100	11091 20317	05694 09633	08s0007g01940	4g015260 7g067340	006s09880 016s12010
						3DS_CBE46365A								
						2BL_4CF076AAB								
						2AL_218CD1AAD								
						3B_4DEB64E2E								
***ZAT6***	5g04340	03g32230	106026	01g031900	1g16010	4BS_FE945DFC	54674	17G35430	01g107170	34322	09464	03s0091g00690	1g106730	002s12010
					2g49250	4DS_98E9655C8								
					4g03340	4DS_676CACEAC	51405							
							70662							

The table is mainly produced using *Arabidopsis* gene ids as the reference and compiling their orthologs from the Plant ensembl database [173]. Some gene ids are adopted from Table 1 of [174]. ND: No data, (*) miR399 orthologs are taken from miRBase database [175], (**) is the RAP id and does not include prefix like MSU id of other rice genes.

## Supplementary Materials

The following are available online at https://www.mdpi.com/2073-4425/10/2/139/s1, Supplementary Information File S1.

## References

[1] Ray D.K., Mueller N.D., West P.C., Foley J.A. (2013). Yield trends are insufficient to double global crop production by 2050. PLoS ONE.

[2] Godfray H.C.J., Beddington J.R., Crute I.R., Haddad L., Lawrence D., Muir J.F., Pretty J., Robinson S., Thomas S.M., Toulmin C. (2010). Food security: The challenge of feeding 9 billion people. Science.

[3] Gregory P.J., George T.S. (2011). Feeding nine billion: The challenge to sustainable crop production. J. Exp. Bot..

[4] Ray D.K., Ramankutty N., Mueller N.D., West P.C., Foley J.A. (2012). Recent patterns of crop yield growth and stagnation. Nat. Commun..

[5] Bruinsma J. (2017). World Agriculture: Towards 2015/2030: An FAO Perspective.

[6] Gilbert N. (2009). Environment: The disappearing nutrient. Nat. News.

[7] Fixen P.E., Johnston A.M. (2012). World fertilizer nutrient reserves: A view to the future. J. Sci. Food Agric..

[8] Johnston A.E., Poulton P.R., Fixen P.E., Curtin D. (2014). Phosphorus: Its efficient use in agriculture. Advances in Agronomy.

[9] Elser J.J. (2012). Phosphorus: A limiting nutrient for humanity?. Curr. Opin. Biotechnol..

[10] Johnston A., Steen I. (2000). Understanding Phosphorus and Its Use in Agriculture.

[11] Syers J., Johnston A., Curtin D. (2008). Efficiency of Soil and Fertiliser Phosphorus Use: Reconciling Changing Concepts of Soil Phosphorus Behaviour with Agronomic Information.

[12] Faucon M.-P., Houben D., Reynoird J.-P., Mercadal-Dulaurent A.-M., Armand R., Lambers H. (2015). Advances and perspectives to improve the phosphorus availability in cropping systems for agroecological phosphorus management. Advances in Agronomy.

[13] Gaxiola R.A., Edwards M., Elser J.J. (2011). A transgenic approach to enhance phosphorus use efficiency in crops as part of a comprehensive strategy for sustainable agriculture. Chemosphere.

[14] Fischer R.A., Connor D.J. (2018). Issues for cropping and agricultural science in the next 20 years. Field Crop. Res..

[15] Maathuis F.J. (2009). Physiological functions of mineral macronutrients. Curr. Opin. Plant Boil..

[16] Mehra P., Pandey B.K., Verma L., Giri J. (2018). A Novel Glycerophosphodiester Phosphodiesterase Improves Phosphate Deficiency Tolerance. Plant Cell Environ..

[17] Schachtman D.P., Reid R.J., Ayling S.M. (1998). Phosphorus uptake by plants: From soil to cell. Plant Physiol..

[18] Pierre W., Parker F. (1927). Soil phosphorus studies: II. The concentration of organic and inorganic phosphorus in the soil solution and soil extracts and the availability of the organic phosphorus to plants. Soil Sci..

[19] Raghothama K. (1999). Phosphate acquisition. Annu. Rev. Plant Boil..

[20] Pratt J., Boisson A.-M., Gout E., Bligny R., Douce R., Aubert S. (2009). Phosphate (Pi) starvation effect on the cytosolic Pi concentration and Pi exchanges across the tonoplast in plant cells: An in vivo 31P-nuclear magnetic resonance study using methylphosphonate as a Pi analog. Plant Physiol..

[21] Lambers H., Plaxton W.C. (2015). Phosphorus: Back to the Roots. Annu. Plant Rev..

[22] Mehra P., Pandey B., Giri J. (2017). Improvement in phosphate acquisition and utilization by a secretory purple acid phosphatase (OsPAP21b) in rice. Plant Biotechnol. J..

[23] Foyer C., Spencer C. (1986). The relationship between phosphate status and photosynthesis in leaves. Planta.

[24] Kennelly M., O’Mara J., Rivard C., Miller G.L., Smith D. (2012). Introduction to abiotic disorders in plants. Plant Health Instr..

[25] Hernández I., Munné-Bosch S. (2015). Linking phosphorus availability with photo-oxidative stress in plants. J. Exp. Bot..

[26] Smith F.W. (2002). The phosphate uptake mechanism. Food Security in Nutrient-Stressed Environments: Exploiting Plants’ Genetic Capabilities.

[27] Jeschke W.D., Kirkby E.A., Peuke A.D., Pate J.S., Hartung W. (1997). Effects of P deficiency on assimilation and transport of nitrate and phosphate in intact plants of castor bean (*Ricinus communis* L.). J. Exp. Bot..

[28] Jouhet J., Maréchal E., Baldan B., Bligny R., Joyard J., Block M.A. (2004). Phosphate deprivation induces transfer of DGDG galactolipid from chloroplast to mitochondria. J. Cell Biol..

[29] Zhang Z., Liao H., Lucas W.J. (2014). Molecular mechanisms underlying phosphate sensing, signaling, and adaptation in plants. J. Integr. Plant Boil..

[30] Hammond J.P., Bennett M.J., Bowen H.C., Broadley M.R., Eastwood D.C., May S.T., Clive R., Ranjan S., Woolaway K.E., White P.J. (2003). Changes in gene expression in *Arabidopsis* shoots during phosphate starvation and the potential for developing smart plants. Plant Physiol..

[31] Pandey B.K., Mehra P., Verma L., Bhadouria J., Giri J. (2017). OsHAD1, a Haloacid Dehalogenase-Like APase, Enhances Phosphate Accumulation. Plant Physiol..

[32] Niu Y.F., Chai R.S., Jin G.L., Wang H., Tang C.X., Zhang Y.S. (2013). Responses of root architecture development to low phosphorus availability: A review. Ann. Bot..

[33] Richardson A.E., Lynch J.P., Ryan P.R., Delhaize E., Smith F.A., Smith S.E., Harvey P.R., Ryan M.H., Veneklaas E.J., Lambers H. (2011). Plant and microbial strategies to improve the phosphorus efficiency of agriculture. Plant Soil.

[34] Benjamin P., Mathilde C., Laurent N., Thierry D. (2011). Root developmental adaptation to phosphate starvation: Better safe than sorry. Trends Plant Sci..

[35] Haling R.E., Brown L.K., Stefanski A., Kidd D.R., Ryan M.H., Sandral G.A., George T.S., Lambers H., Simpson R.J. (2018). Differences in nutrient foraging among *Trifolium subterraneum* cultivars deliver improved P-acquisition efficiency. Plant Soil.

[36] Jung J.K.H., Susan M.C. (2013). Getting to the roots of it: Genetic and hormonal control of root architecture. Front. Plant Sci..

[37] Lai F., Jennifer T., Li Y., Peter D. (2007). Cell division activity determines the magnitude of phosphate starvation responses in *Arabidopsis*. Plant J..

[38] Ticconi C.A., Lucero R.D., Sakhonwasee S., Adamson A.W., Creff A., Nussaume L., Desnos T., Abel S., Amasino R.M. (2009). ER-Resident Proteins PDR2 and LPR1 Mediate the Developmental Response of Root Meristems to Phosphate Availability. Proc. Natl. Acad. Sci. USA.

[39] Sánchez-Calderón L., López-Bucio J., Chacón-López A., Cruz-Ramírez A., Nieto-Jacobo F., Dubrovsky J.G., Herrera-Estrella L. (2005). Phosphate starvation induces a determinate developmental program in the roots of *Arabidopsis thaliana*. Plant Cell Physiol..

[40] López-Bucio J., Hernández-Abreu E., Sánchez-Calderón L., Nieto Jacobo M., Simpson J., Herrera-Estrella L. (2002). Phosphate availability alters architecture and causes changes in hormone sensitivity in the *Arabidopsis* root system. Plant Physiol..

[41] Williamson L.C., Ribrioux S.P.C.P., Fitter A.H., Leyser H.M.O. (2001). Phosphate availability regulates root system architecture in *Arabidopsis*. Plant Physiol..

[42] Huang G., Liang W., Sturrock C.J., Pandey B.K., Giri J., Mairhofer S., Wang D., Muller L., Tan H., York L.M. (2018). Rice actin binding protein RMD controls crown root angle in response to external phosphate. Nat. Commun..

[43] Schiefelbein J.W., Somerville C. (1990). Genetic control of root hair development in *Arabidopsis thaliana*. Plant Cell.

[44] Foreman J., Dolan L. (2001). Root Hairs as a Model System for Studying Plant Cell Growth. Ann. Bot..

[45] Ma Z., DG B., Brown K.M., Lynch J.P. (2001). Regulation of root hair density by phosphorus availability in *Arabidopsis thaliana*. Plant Cell Environ..

[46] Bhosale R., Giri J., Pandey B.K. (2018). A mechanistic framework for auxin dependent *Arabidopsis* root hair elongation to low external phosphate. Nat. Commun..

[47] Giri J., Bhosale R., Huang G., Pandey B., Parker H., Zappala S., Yang J., Dievart A., Bureau C., Ljung K. (2018). Rice auxin influx carrier OsAUX1 facilitates root hair elongation in response to low external phosphate. Nat. Commun..

[48] Bates T.R., Lynch J.P. (2000). The efficiency of *Arabidopsis thaliana* (Brassicaceae) root hairs in phosphorus acquisition. Am. J. Bot..

[49] Shane M.W., Lambers H. (2005). Cluster Roots: A Curiosity in Context. Plant Soil.

[50] He C.J., Morgan P.W., Drew M.C. (1992). Enhanced sensitivity to ethylene in nitrogen- or phosphate-starved roots of *Zea mays* L. during aerenchyma formation. Plant Physiol..

[51] Ma Z., Baskin T., Brown K., Lynch J. (2003). Regulation of root elongation under phosphorus stress involves changes in ethylene responsiveness. Plant Physiol..

[52] Javot H., Pumplin N., Harrison M.J. (2007). Phosphate in the arbuscular mycorrhizal symbiosis: transport properties and regulatory roles. Plant Cell Environ..

[53] Dakora F.D., Phillips D.A. (2002). Root exudates as mediators of mineral acquisition in low-nutrient environments. Plant Soil.

[54] Tomscha J.L., Trull M.C., Jill D., Lynch J.P., Guiltinan M.J. (2004). Phosphatase under-producer mutants have altered phosphorus relations. Plant Physiol..

[55] McNear M.N. (2003). The Rhizosphere—Roots, Soil and Everything In Between. Nat. Educ. Knowl..

[56] Czarnecki O., Yang J., Weston D., Tuskan G., Chen J.-G. (2013). A dual role of strigolactones in phosphate acquisition and utilization in plants. Int. J. Mol. Sci..

[57] Smith S.E., Jakobsen I., Grønlund M., Smith F.A. (2011). Roles of arbuscular mycorrhizas in plant phosphorus nutrition: Interactions between pathways of phosphorus uptake in arbuscular mycorrhizal roots have important implications for understanding and manipulating plant phosphorus acquisition. Plant Physiol..

[58] Akiyama K., Hayashi H. (2006). Strigolactones: Chemical signals for fungal symbionts and parasitic weeds in plant roots. Ann. Bot..

[59] Besserer A., Puech-Pages V., Kiefer P., Gomez-Roldan V., Jauneau A., Roy S., Portais J.C., Roux C., Becard G., Sejalon-Delmas N. (2006). Strigolactones stimulate arbuscular mycorrhizal fungi by activating mitochondria. PLoS Biol..

[60] Schweiger P.F., Robson A.D., Barrow N.J. (1995). Root hair length determines beneficial effect of a Glomus species on shoot growth of some pasture species. New Phytol..

[61] Chen W., Li J., Zhu H., Xu P., Chen J., Yao Q. (2017). Arbuscular Mycorrhizal Fungus Enhances Lateral Root Formation in *Poncirus trifoliata* (L.) as Revealed by RNA-Seq Analysis. Front. Plant Sci..

[62] George E., Marschner H., Jakobsen I. (1995). Role of arbuscular mycorrhizal fungi in uptake of phosphorus and nitrogen from soil. Crit. Rev. Biotechnol..

[63] Gutjahr C., Paszkowski U. (2013). Multiple control levels of root system remodeling in arbuscular mycorrhizal symbiosis. Front. Plant Sci..

[64] Svistoonoff S., Creff A., Reymond M., Sigoillot-Claude C., Ricaud L., Blanchet A., Nussaume L., Desnos T. (2007). Root tip contact with low-phosphate media reprograms plant root architecture. Nat. Genet..

[65] Bonnot C., Pinson B., Clément M., Bernillon S., Chiarenza S., Kanno S., Kobayashi N., Delannoy E., Nakanishi T.M., Nussaume L. (2016). A chemical genetic strategy identify the PHOSTIN, a synthetic molecule that triggers phosphate starvation responses in *Arabidopsis thaliana*. New Phytol..

[66] Chiou T.-J., Lin S.-I. (2011). Signaling network in sensing phosphate availability in plants. Annu. Rev. Plant Boil..

[67] Lin W.Y., Huang T.K., Leong S.J., Chiou T.J. (2014). Long-distance call from phosphate: Systemic regulation of phosphate starvation responses. J. Exp. Bot..

[68] Panigrahy M., Rao D.N., Sarla N. (2009). Molecular mechanisms in response to phosphate starvation in rice. Biotechnol. Adv..

[69] Lin S.I., Chiou T.J. (2008). Long-distance movement and differential targeting of microRNA399s. Plant Signal. Behav..

[70] Lin W.Y., Lin S.T. (2009). Molecular regulators of phosphate homeostasis in plants. J. Exp. Bot..

[71] Rubio V., Linhares F., Solano R., Martín A.C., Iglesias J., Leyva A., Pazares J. (2001). A conserved MYB transcription factor involved in phosphate starvation signaling both in vascular plants and in unicellular algae. Genes Dev..

[72] Miura K., Rus A., Sharkhuu A., Yokoi S., Karthikeyan A.S., Raghothama K.G., Baek D., Koo Y.D., Jin J.B., Bressan R.A. (2005). The *Arabidopsis* SUMO E3 ligase SIZ1 controls phosphate deficiency responses. Proc. Natl. Acad. Sci. USA.

[73] Qundan L., Zhong Y., Yuguang W., Wang Z., Li Z., Shi J., Wu Z., Yu L., Mao C., Yi K. (2014). SPX4 negatively regulates phosphate signaling and homeostasis through its interaction with PHR2 in rice. Plant Cell.

[74] María Isabel P., Isabel M., Rajulu C., Zhiye W., Franco-Zorrilla J.M., Laura D.L., Irigoyen M.L., Simona M., Regla B., José R. (2014). SPX1 is a phosphate-dependent inhibitor of Phosphate Starvation Response 1 in *Arabidopsis*. Proc. Natl. Acad. Sci. USA.

[75] Liu T.-Y., Wei-Yi L., Teng-Kuei H., Tzyy-Jen C. (2014). MicroRNA-mediated surveillance of phosphate transporters on the move. Trends Plant Sci..

[76] Briat J.F., Rouached H., Tissot N., Gaymard F., Dubos C. (2015). Integration of P, S, Fe, and Zn nutrition signals in *Arabidopsis thaliana*: Potential involvement of PHOSPHATE STARVATION RESPONSE 1 (PHR1). Front. Plant Sci..

[77] Pant B.D., Burgos A., Pant P., Cuadrosinostroza A., Willmitzer L., Scheible W. (2015). The transcription factor PHR1 regulates lipid remodeling and triacylglycerol accumulation in *Arabidopsis thaliana* during phosphorus starvation. J. Exp. Bot..

[78] Plaxton W.C., Lambers H. (2015). ’Omics’ Approaches Towards Understanding Plant Phosphorus Acquisition and Use.

[79] Plaxton W.C., Shane M.W. (2015). The Role of Post-Translational Enzyme Modifications in the Metabolic Adaptations of Phosphorus-Deprived Plants. Annual Plant Reviews.

[80] Secco D., Wang C., Shou H., Schultz M.D., Chiarenza S., Nussaume L., Ecker J.R., Whelan J., Lister R. (2015). Stress induced gene expression drives transient DNA methylation changes at adjacent repetitive elements. Elife.

[81] David S., Mehdi J., Hayden W., Huixia S., Ping W., Yves P., James W. (2013). Spatio-temporal transcript profiling of rice roots and shoots in response to phosphate starvation and recovery. Plant Signal. Behav..

[82] Yong-Villalobos L., Cervantes-Pérez S.A., Gutiérrez-Alanis D., Gonzáles-Morales S., Martínez O., Herrera-Estrella L. (2016). Phosphate starvation induces DNA methylation in the vicinity of *cis*-acting elements known to regulate the expression of phosphate-responsive genes. Plant Signal. Behav..

[83] Plaxton W.C., Tran H.T. (2011). Metabolic adaptations of phosphate-starved plants. Plant Physiol..

[84] Plaxton W.C., Lambers H. (2015). Metabolomics of Plant Phosphorus-Starvation Response. Annual Plant Reviews.

[85] Tudzynski B. (2001). Plant Responses to Environmental Stresses: From Phytohormones to Genome Reorganization: H.R. Lerner (Ed.). Marcel Dekker, New York, Basel, 1999, 730 pp., ISBN 0-8247-0044-9. Phytochemistry.

[86] Morcuende R., Bari R., Gibon Y., Zheng W., Pant B.D., Bl Sing O., Usadel B.R., Czechowski T., Udvardi M.K., Stitt M. (2010). Genome-wide reprogramming of metabolism and regulatory networks of *Arabidopsis* in response to phosphorus. Plant Cell Environ..

[87] Pant B.D., Pant P., Erban A., Huhman D., Kopka J., Scheible W.R. (2015). Identification of primary and secondary metabolites with phosphorus status-dependent abundance in *Arabidopsis*, and of the transcription factor PHR1 as a major regulator of metabolic changes during phosphorus limitation. Plant Cell Environ..

[88] ricexPro.dna.affrc.go.jp. http://ricexpro.dna.affrc.go.jp/RXP_4001/index.php.

[89] Hammond J.P., White P.J. (2008). Sucrose transport in the phloem: Integrating root responses to phosphorus starvation. J. Exp. Bot..

[90] Hermans C., Hammond J.P., White P.J., Verbruggen N. (2006). How do plants respond to nutrient shortage by biomass allocation?. Trends Plant Sci..

[91] Solfanelli C., Perata P. (2006). Sucrose-Specific Induction of the Anthocyanin Biosynthetic Pathway in *Arabidopsis*. Plant Physiol..

[92] Karthikeyan A., Varadarajan D., Jain A., Held M., Carpita N., Raghothama K. (2007). Phosphate starvation responses are mediated by sugar signaling in *Arabidopsis*. Planta.

[93] Hammond J.P., Broadley M.R., White P.J. (2004). Genetic responses to phosphorus deficiency. Ann. Bot..

[94] Lloyd J.C., Zakhleniuk O.V. (2004). Responses of primary and secondary metabolism to sugar accumulation revealed by microarray expression analysis of the *Arabidopsis* mutant, pho3. J. Exp. Bot..

[95] Müller R., Morant M., Jarmer H., Nilsson L., Nielsen T.H. (2007). Genome-Wide Analysis of the *Arabidopsis* Leaf Transcriptome Reveals Interaction of Phosphate and Sugar Metabolism. Plant Physiol..

[96] Ticconi C.A., Abel S. (2004). Short on phosphate: Plant surveillance and countermeasures. Trends Plant Sci..

[97] Hernández G., Oswaldo V.L., Mario R., Nicolas G., Georg W., Rosaura A.F., Sara Isabel F., Alexander E., Joachim K., Udvardi M.K. (2009). Global changes in the transcript and metabolic profiles during symbiotic nitrogen fixation in phosphorus-stressed common bean plants. Plant Physiol..

[98] Obata T., Fernie A.R. (2012). The use of metabolomics to dissect plant responses to abiotic stresses. Cell. Mol. Life Sci..

[99] Lahner B., Gong J.M., Smith E.L., Abid K.B., Rogers E.E. (2003). Genomic scale profiling of nutrient and trace elements in *Arabidopsis thaliana*. Nat. Biotechnol..

[100] Salt D.E. (2004). Update on plant ionomics. Plant Physiol..

[101] Baxter I. (2009). Ionomics: Studying the social network of mineral nutrients. Curr. Opin. Plant Biol..

[102] Baxter I., Springer N., Jackson S. (2010). Ionomics: The functional genomics of elements. Brief. Funct. Genom..

[103] Conn S., Gilliham M. (2010). Comparative physiology of elemental distributions in plants. Ann. Bot..

[104] Baxter I.R., Vitek O., Lahner B., Muthukumar B., Borghi M., Morrissey J., Guerinot M.L., Salt D.E. (2008). The leaf ionome as a multivariable system to detect a plant’s physiological status. Proc. Natl. Acad. Sci. USA.

[105] Schlüter U., Colmsee C., Scholz U., Bräutigam A., Weber A.P., Zellerhoff N., Bucher M., Fahnenstich H., Sonnewald U. (2013). Adaptation of maize source leaf metabolism to stress related disturbances in carbon, nitrogen and phosphorus balance. BMC Genom..

[106] Naoko O.O., Jun W. (2010). Recent progress in plant nutrition research: Cross-talk between nutrients, plant physiology and soil microorganisms. Plant Cell Physiol..

[107] Baxter I., Dilkes B.P. (2012). Elemental profiles reflect plant adaptations to the environment. Science.

[108] Dai X., Wang Y., Zhang W.-H. (2015). OsWRKY74, a WRKY transcription factor, modulates tolerance to phosphate starvation in rice. J. Exp. Bot..

[109] Kawa D., Julkowska M., Montero Sommerfeld H., ter Horst A., Haring M.A., Testerink C. (2016). Phosphate-dependent root system architecture responses to salt stress. Plant Physiol..

[110] Wild R., Gerasimaite R., Jung J.Y., Truffault V., Pavlovic I., Schmidt A., Saiardi A., Jessen H.J., Poirier Y., Hothorn M. (2016). Control of eukaryotic phosphate homeostasis by inositol polyphosphate sensor domains. Science.

[111] Yue W., Ying Y., Wang C., Zhao Y., Dong C., Whelan J., Shou H. (2017). OsNLA1, a RING-type ubiquitin ligase, maintains phosphate homeostasis in *Oryza sativa* via degradation of phosphate transporters. Plant J. Cell Mol. Boil..

[112] Simpson R.J., Stefanski A., Marshall D.J., Moore A.D., Richardson A.E. (2015). Management of soil phosphorus fertility determines the phosphorus budget of a temperate grazing system and is the key to improving phosphorus efficiency. Agric. Ecosyst. Environ..

[113] Jia X., Liu P., Lynch J.P. (2018). Greater lateral root branching density in maize improves phosphorus acquisition from low phosphorus soil. J. Exp. Bot..

[114] Galindo-Castaneda T., Brown K.M., Lynch J.P. (2018). Reduced root cortical burden improves growth and grain yield under low phosphorus availability in maize. Plant Cell Environ..

[115] Strock C.F., Morrow de la Riva L., Lynch J.P. (2018). Reduction in Root Secondary Growth as a Strategy for Phosphorus Acquisition. Plant Physiol..

[116] Rose T., Liu L., Wissuwa M. (2013). Improving phosphorus efficiency in cereal crops: Is breeding for reduced grain phosphorus concentration part of the solution?. Front. Plant Sci..

[117] Ha S., Tran L.S. (2014). Understanding plant responses to phosphorus starvation for improvement of plant tolerance to phosphorus deficiency by biotechnological approaches. Crit. Rev. Biotechnol..

[118] Lópezarredondo D.L., Leyvagonzález M.A., Gonzálezmorales S.I., Lópezbucio J., Herreraestrella L. (2014). Phosphate Nutrition: Improving Low-Phosphate Tolerance in Crops. Annu. Rev. Plant Boil..

[119] Lambers H., Finnegan P.M., Jost R., Plaxton W.C., Shane M.W., Stitt M. (2015). Phosphorus nutrition in Proteaceae and beyond. Nat. Plants.

[120] Brown L.K., George T.S., Dupuy L.X., White P.J. (2013). A conceptual model of root hair ideotypes for future agricultural environments: What combination of traits should be targeted to cope with limited P availability?. Ann. Bot..

[121] Scheible W.R., Rojas-Triana M. (2015). Sensing, Signaling and Control of Phosphate Starvation in Plants: Molecular Players and Applications. Annual Plant Reviews.

[122] Vance C.P. (2010). Quantitative trait loci, epigenetics, sugars, and microRNAs: Quaternaries in phosphate acquisition and use. Plant Physiol..

[123] Chin J.H., Rico G., Cheryl D., Masdiar B., Joko P., Sugiono M., Matthias W., Sigrid H. (2011). Developing rice with high yield under phosphorus deficiency: Pup1 sequence to application. Plant Physiol..

[124] Lynch J.P. (2011). Root phenes for enhanced soil exploration and phosphorus acquisition: Tools for future crops. Plant Physiol..

[125] Wissuwa M., Wegner J., Ae N., Yano M. (2002). Substitution mapping of Pup1: A major QTL increasing phosphorus uptake of rice from a phosphorus-deficient soil. Theor. Appl. Genet..

[126] Gamuyao R., Chin J.H., Pariascatanaka J., Pesaresi P., Catausan S., Dalid C., Slametloedin I., Tecsonmendoza E.M., Wissuwa M., Heuer S. (2012). The protein kinase Pstol1 from traditional rice confers tolerance of phosphorus deficiency. Nature.

[127] Richardson A.E. (2009). Regulating the phosphorus nutrition of plants: Molecular biology meeting agronomic needs. Plant Soil.

[128] Mittler R., Blumwald E. (2010). Genetic engineering for modern agriculture: Challenges and perspectives. Annu. Rev. Plant Boil..

[129] Ramaekers L., Remans R., Rao I.M., Blair M.W., Vanderleyden J. (2010). Strategies for improving phosphorus acquisition efficiency of crop plants. Field Crop. Res..

[130] Tian J., Xiurong W., Yiping T., Xinping C., Hong L. (2012). Bioengineering and management for efficient phosphorus utilization in crops and pastures. Curr. Opin. Biotechnol..

[131] Pérez-Clemente R.M., Vives V., Zandalinas S.I., López-Climent M.F., Muñoz V., Gómez-Cadenas A. (2013). Biotechnological approaches to study plant responses to stress. BioMed Res. Int..

[132] Péret B., Li G., Zhao J., Band L.R., Voß U., Postaire O., Luu D.T., Da Ines O., Casimiro I., Lucas M. (2012). Auxin regulates aquaporin function to facilitate lateral root emergence. Nat. Cell Biol..

[133] Marbach D., Costello J., Küffner R., Vega N., Prill R., Camacho D., Allison K., Kellis M., Collins J., Stolovitzky G. (2012). Wisdom of crowds for robust gene network inference. Nat. Methods.

[134] Pan Y., Glyn B., Kevin P., Graham B., Chungui L., Rupert F., Alexandra M., Subhalai J., Charles B., Rik V.W. (2013). Network inference analysis identifies an APRR2-like gene linked to pigment accumulation in tomato and pepper fruits. Plant Physiol..

[135] Tak L., Taeyun O., Sunmo Y., Junha S., Sohyun H., Yeong K.C., Hyojin K., Hongseok S., Jung Eun S., Ronald P.C. (2015). RiceNet v2: An improved network prioritization server for rice genes. Nucleic Acids Res..

[136] Lucas M., Laplaze L., Bennett M.J. (2011). Plant Systems Biology: Network Matters. Plant Cell Environ..

[137] Lavenus J., Middleton A., Wilson M., Lucas M., Laplaze L., Bennett M., Crespi M. (2012). Toward a Virtual Root: Interaction of Genomics and Modeling to Develop Predictive Biology Approaches. Root Genomics and Soil Interactions.

[138] Kristine H., Silvana P., Guillaume L., Susan Z., Sacha M., Xavier D., Bennett M.J. (2013). Root Systems Biology: Integrative Modeling across Scales, from Gene Regulatory Networks to the Rhizosphere. Plant Physiol..

[139] Hodgman T.C., Ajmera I. (2015). The successful application of systems approaches in plant biology. Prog. Biophys. Mol. Biol..

[140] Tardieu F., Tardieu F. (2010). Why work and discuss the basic principles of plant modelling 50 years after the first plant models?. J. Exp. Bot..

[141] Idso S.B. (1978). Mathematical Models in Plant Physiology: A Quantitative Approach to Problems in Plant and Crop Physiology: J. H. M. Thornley. Experimental Botany, Vol. 8, Academic Press, London, 1976, 318 pp., £9.80.

[142] Ajmera I., Shi J., Giri J., Wu P., Stekel D.J., Lu C., Hodgman T.C. (2018). Regulatory feedback response mechanisms to phosphate starvation in rice. NPJ Syst. Boil. Appl..

[143] Roose T., Fowler A.C. (2004). A mathematical model for water and nutrient uptake by plant root systems. J. Theor. Boil..

[144] Leitner D., Klepsch S., Ptashnyk M., Marchant A., Kirk G.J., Schnepf A., Roose T. (2010). A dynamic model of nutrient uptake by root hairs. New Phytol..

[145] Ptashnyk M., Roose T., Jones D.L., Kirk G.J. (2011). Enhanced zinc uptake by rice through phytosiderophore secretion: A modelling study. Plant Cell Environ..

[146] Zygalakis K.C., Kirk G.J.D., Jones D.L., Wissuwa M., Roose T. (2011). A dual porosity model of nutrient uptake by root hairs. New Phytol..

[147] Zygalakis K.C., Roose T. (2012). A mathematical model for investigating the effect of cluster roots on plant nutrient uptake. Eur. Phys. J. Spec. Top..

[148] Keyes S.D., Daly K.R., Gostling N.J., Jones D.L., Talboys P., Pinzer B.R., Boardman R., Sinclair I., Marchant A., Roose T. (2013). High resolution synchrotron imaging of wheat root hairs growing in soil and image based modelling of phosphate uptake. New Phytol..

[149] Payvandi S., Daly K.R., Jones D.L., Talboys P., Zygalakis K.C., Roose T. (2014). A Mathematical Model of Water and Nutrient Transport in Xylem Vessels of a Wheat Plant. Bull. Math. Boil..

[150] Heppell J., Payvandi S., Talboys P., Zygalakis K.C., Fliege J., Langton D., Sylvester-Bradley R., Walker R., Jones D.L., Roose T. (2016). Modelling the optimal phosphate fertiliser and soil management strategy for crops. Plant Soil.

[151] Andrea S., Tiina R. (2010). Modelling the contribution of arbuscular mycorrhizal fungi to plant phosphate uptake. New Phytol..

[152] Schnepf A., Roose T., Schweiger P. (2008). Impact of growth and uptake patterns of arbuscular mycorrhizal fungi on plant phosphorus uptake—A modelling study. Plant Soil.

[153] Schnepf A., Jones D., Roose T. (2011). Modelling Nutrient Uptake by Individual Hyphae of Arbuscular Mycorrhizal Fungi: Temporal and Spatial Scales for an Experimental Design. Bull. Math. Biol..

[154] Kirk G.J.D. (2002). Modelling root-induced solubilization of nutrients. Plant Soil.

[155] Barrow N.J. (1983). A mechanistic model for describing the sorption and desorption of phosphate by soil. J. Soil Sci..

[156] Silberbush M., Barber S.A. (1983). Sensitivity of simulated phosphorus uptake to parameters used by a mechanistic-mathematical model. Plant Soil.

[157] Grant R.F., Robertson J.A. (1997). Phosphorus uptake by root systems: Mathematical modelling in ecosys. Plant Soil.

[158] Rowell D.L. (2010). Solute Movement in the Rhizosphere. J. Soil Sci..

[159] Amijee F., Barraclouch P.B., Tinker P.B. (1991). Modeling phosphorus uptake and utilization by plants. Phosphorus Nutrition of Grain Legumes in the Semi-Arid Tropics.

[160] Matthias W. (2003). How do plants achieve tolerance to phosphorus deficiency? Small causes with big effects. Plant Physiol..

[161] Heppell J., Talboys P., Payvandi S., Zygalakis K.C., Fliege J., Withers P.J., Jones D.L., Roose T. (2015). How changing root system architecture can help tackle a reduction in soil phosphate (P) levels for better plant P acquisition. Plant Cell Environ..

[162] Postma J.A., Lynch J.P. (2011). Theoretical evidence for the functional benefit of root cortical aerenchyma in soils with low phosphorus availability. Ann. Bot..

[163] Richard H., Claire H., Penfold C.A., Emily B., Laura B., Moore J.D., Peijun Z., Alison J., Emma C., Findlay B.C. (2013). A local regulatory network around three NAC transcription factors in stress responses and senescence in *Arabidopsis* leaves. Plant J. Cell Mol. Biol..

[164] Brown T.B., Cheng R., Sirault X.R.R., Rungrat T., Murray K.D., Trtilek M., Furbank R.T., Badger M., Pogson B.J., Borevitz J.O. (2014). TraitCapture: Genomic and environment modelling of plant phenomic data. Curr. Opin. Plant Biol..

[165] Veneklaas E., Lambers H., Bragg J., Finnegan P., Lovelock C., Plaxton W., Price C., Scheible W., Shane M., White P. (2012). Opportunities for improving phosphorus-use efficiency in crop plants. New Phytol..

[166] Christophe P., Samuel D., Frédéric B., Christian F., Christophe G. (2008). OpenAlea: A visual programming and component-based software platform for plant modelling. Funct. Plant Biol..

[167] Merks R., Guravage M., Inzé D., Beemster G. (2011). Virtualleaf: An open-source framework for cell-based modeling of plant tissue growth and development. Plant Physiol..

[168] Hoon C.Y., Bénédicte W., Anna F., Virginie M., Jasper T., Davey C.L., Christopher T., Howard T., Ougham H.J., Philippe D.R. (2014). Multiscale digital *Arabidopsis* predicts individual organ and whole-organism growth. Proc. Natl. Acad. Sci. USA.

[169] Arnaud C., Clement M., Thibaud M., Javot H., Chiarenza S., Delannoy E., Revol J., Soreau P., Bo C., Block M. (2014). Identification of Phosphatin, a drug alleviating phosphate starvation responses in *Arabidopsis*. Plant Physiol..

[170] Ricarda J., Made P., Lapis-Gaza H.R., Claudia R., Oliver B., Hans L., Finnegan P.M. (2015). Differentiating phosphate-dependent and phosphate-independent systemic phosphate-starvation response networks in *Arabidopsis thaliana* through the application of phosphite. J. Exp. Bot..

[171] Fang Z., Shao C., Meng Y., Wu P., Chen M. (2009). Phosphate signaling in *Arabidopsis* and *Oryza sativa*. Plant Sci..

[172] Carlos C.V., Sawers R.J.H., Luis H.E. (2011). Phosphate deprivation in maize: Genetics and genomics. Plant Physiol..

[173] plant.ensembl.org. http://plants.ensembl.org/index.html.

[174] Calderon-Vazquez C., Sawers J.H.R., Herrera-Estrella L. (2011). Phosphate Deprivation in Maize: Genetics and Genomics. Plant Physiology..

[175] Mirbase.org. http://mirbase.org.

[176] Wilson M.H., Holman T.J., Sørensen I., Canchosanchez E., Wells D.M., Swarup R., Knox J.P., Willats W.G., Ubedatomás S., Holdsworth M. (2015). Multi-omics analysis identifies genes mediating the extension of cell walls in the *Arabidopsis thaliana* root elongation zone. Front. Cell Dev. Biol..

[177] Band L.R., Fozard J.A., Christophe G., Jensen O.E., Tony P., Bennett M.J., King J.R. (2012). Multiscale systems analysis of root growth and development: Modeling beyond the network and cellular scales. Plant Cell.

[178] Yang S.Y., Huang T.K., Kuo H.F., Chiou T.J. (2017). Role of vacuoles in phosphorus storage and remobilization. J. Exp. Bot..

[179] Mukherjee P., Banerjee S., Wheeler A., Ratliff L.A., Irigoyen S., Garcia L.R., Lockless S.W., Versaw W.K. (2015). Live imaging of inorganic phosphate in plants with cellular and subcellular resolution. Plant Physiol..

[180] De Col V., Fuchs P., Nietzel T., Elsasser M., Voon C.P., Candeo A., Seeliger I., Fricker M.D., Grefen C., Moller I.M. (2017). ATP sensing in living plant cells reveals tissue gradients and stress dynamics of energy physiology. Elife.

[181] Chapin F.S. (1991). Effects of multiple environmental stresses on nutrient availability and use. Response of Plants to Multiple Stresses.

[182] Kenji M., Aiko S., Masaru O., Jun F. (2011). Increased tolerance to salt stress in the phosphate-accumulating *Arabidopsis* mutants *siz1* and *pho2*. Planta.

[183] Plaxton W.C., Lambers H. (2015). Interactions between Nitrogen and Phosphorus Metabolism. Annual Plant Reviews.

[184] Giehl R.F.H., Gruber B.D., Von Wirén N. (2014). It’s time to make changes: Modulation of root system architecture by nutrient signals. J. Exp. Bot..

[185] Vos J., Evers J.B., Bucksorlin G.H., Andrieu B., Chelle M., Visser P.H.B.D., Tardieu F. (2010). Functional-structural plant modelling: A new versatile tool in crop science. J. Exp. Bot..

[186] Kuijken R.C., van Eeuwijk F.A., Marcelis L.F., Bouwmeester H.J. (2015). Root phenotyping: From component trait in the lab to breeding. J. Exp. Bot..

[187] Das A., Schneider H., Burridge J., Ascanio A.K.M., Wojciechowski T., Topp C.N., Lynch J.P., Weitz J.S., Bucksch A. (2015). Digital imaging of root traits (DIRT): A high-throughput computing and collaboration platform for field-based root phenomics. Plant Methods.

