# Supplementary material for: An Integrative Systems Perspective on Plant Phosphate Research
Source: Genes (Basel). 2019 Feb 13;10(2):139. doi: 10.3390/genes10020139 (PMC6410211; doi:10.3390/genes10020139)
Supplement: Supplementary file 1 [file genes-10-00139-s001.pdf]

I. Current strategies at global, national and field scale

Of the total phosphorus used for global food production annually, only 20% actually becomes part of the food consumed, while the majority is lost permanently or temporarily at various stages from mine to fork (Schröder *et al.*, 2010; Cordell & White, 2013). Such considerable losses offer substantial opportunities for improving efficiency of phosphorus management systems, recycling and reuse and enhancing uptake and use efficiency by plant. These opportunities operate at different scales; global, national, local, field and plant scales. The existing strategies and practices utilising these opportunities are discussed as follows.

A. Global scale:

Due to the inequitable geographical distribution of phosphorus reserves, countries dependent on imports are concern over geo-political dynamics of phosphorus. Despite phosphorus being essential to food production, there are no adequate international actions to ensure its access for long-term food security. Phosphate prices hiked in 2008 by 800% putting the issue of global phosphate scarcity into the mainstream international agenda (Cordell *et al.*, 2009). Whereby, there has been increasing support, awareness and advocacy for sustainable management of phosphate. Apart from isolated examples in a few countries, at the international level there are a lack strategic frameworks and policies to stimulate and support the development and efficient use of renewable phosphorus fertilisers and their efficient use and equitable access by all the world's farmers. Besides these, there is a lack of consensus on the various subjects, including the life expectancy of global phosphate rock reserves. There is an ambiguity of roles and absence of responsibilities among those organisations who could take the lead on phosphorus security. These include the UN's FAO and UNEP, the fertiliser industry and national governments. Currently by default, the market governs the global phosphate resources, but it alone could hardly solve the rising problem in a sustainable, equitable, and timely manner. Taking the initiative, researchers are forming new global and national platforms, such as the Global Phosphorus Research Initiative (GPRI), European Sustainable Phosphorus Platform (ESPP) and the Dutch Nutrient Platform to address this global issue. Perhaps, it is high time to form an Intergovernmental Panel on Phosphorus Security (SPS committee 2012).

B. National and Field scale:

This scale encompasses different sectors of the food system, from mining to food consumption and wastewater. Broadly, the strategies across these sectors either aim to reduce the demand, or secure supply.

i. Demand reduction

Proper management of storage and application methods, rate, time and place of fertilisers, would minimise P losses and the consequent environmental risks. Different phosphorus management strategies are required for different soil, environment and crops types across the world. One common management criterion is to maintain critical phosphorus levels by replenishing the soil at same rate that it is being removed (Cordell & White, 2013).

To limit the application and immobilisation of phosphate in soil, there are increasing incentives for replacing the 'fixed insurance' method by a 'precision farming' application. Such targeted applications (e.g. near to the roots or intensely rooted part of a soil, as a seed dressing, or foliage feed) are more

economic and offer less risk to the environment (Withers *et al.*, 2014; Withers *et al.*, 2015). Other P fertiliser placement methods include reduced application for better utilisation of legacy soil P, sub-surface banding near to the seed row, and shallow incorporation on a vertical plane with appropriate spreading pattern horizontally (Simpson *et al.*, 2011)**.** Further, to improve the efficiency of phosphorus fertiliser, a number of commercial formulations are available that reduce P immobilisation and enhance the mobilisation of native fixed P. These include polymer-coated organo-mineral and liquid product for soil applications and bioinoculants (McLaughlin *et al.*, 2011). The use of slow-release fertilisers and/or targeted sub-surface application can reduce incidental P loss and environmental risk.

The loss of soil phosphorus from farms results from erosion by water, wind, tillage and harvesting. The infiltration capacity or structural quality of the soils can be improved by various combinations of techniques including, mulching, ridge tillage, sub-soiling, contour ploughing, buffer stripping, cover crop establishment, conversion of arable land into grass land and agroforestry (Cordell & White, 2013). However, the optimal combinations have to be determined for each field individually.

A good level of biodiversity also influences the availability and P uptake by plants. Given this fact, various farm soils are inoculated with microbes to improve P availability (Sharma *et al.*, 2013). For example, Plant Growth Promoting Rhizobacteria (PGPR) directly increases plant available P by solubilisation and mineralisation of fixed P (Ahemad & Kibret, 2014). Another rhizobacteria (e.g. *Azospirillum*), indirectly enhances plant P acquisition by production of phytohormones that stimulate root growth. The commercial inoculants of PGPR have been developed but their widespread application has remained limited due to the inconsistent performance in different environment and poor understanding of associated mechanisms (Simpson *et al.*, 2011) . On similar lines, there are commercial chemical formulations for application to soil, which activate plant gene/s that enhance soil P availability, its uptake and ultimate crop productivity. However, this strategy has remained expensive for the farmers.

The awareness of these strategies is increasing slowly among the farmer communities of the developing world. In some countries, mechanisms have been devised for encouraging farmers to manage phosphate more effectively in area at risk from phosphorus pollution. For example, the UK have several initiatives including Catchment sensitive Farming (CSF), Tried and tested nutrient management and SWARM knowledge hub, to aid farmers in reducing P pollution in agriculture. Between 2006 and 2014, the CSF scheme reduced agricultural P losses on average by ~9% (total phosphorus) and ~7% (reactive phosphorus) (Wentworth, 2014)

As livestock utilise only a small fraction of organic phosphate present in plant components, inorganic phosphate supplements are added to their feed, which account for 5% of the global phosphate supply. A high proportion of total phosphorus ends up in manure. In countries, like Denmark and the Netherlands, an artificial enzyme, 'phytase', is added to the pig feed to breakdown phosphate compounds to a form that is easily absorbed (SCU, 2013). In Canada, genetically-engineered pig 'Enviropig' has been developed that can directly digest the phosphate, thereby reducing the need for phosphate supplements and its level in manure (Forsberg *et al.*, 2003).

Human dietary shift toward phosphorus intensive products has a large contribution in amplifying global P cycle. Compared to vegetarian diet, production of meat and dairy food requires more land and fertilisers, ~ 3 times more phosphate. Meat consumption accounts for 72% of the global average P footprint (Geneviève, 2012) thereby challenging sustainable management of P. Certainly, societal initiative to promote less phosphorus intensive food products i.e. vegetal base, could play an important role, particularly in high P footprint countries, in reducing phosphorus demand along with other human health and environment issues.

Approximately, 55% of phosphorus in food for human consumption is lost as waste during processing, transportation and storage (Cordell *et al.*, 2009). In 2013, it was estimated that total annual food waste rose to ~12 million tonnes within the UK (valued over £19bn and 20mt greenhouse gas emission). Three quarter of this could have been avoided. Decreasing waste in food production and consumption chain would obviously reduce P demand (Cordell & White, 2013).

Food additives comprise 5-10% of the P in domestic waste water in the UK. As only a few alternatives to P-based additives have been licensed for food production, the Environment Agency has initiated the work to find potential alternatives. P compounds also form a core component of detergents, accounting for ~ 10% of the globally mined P. As one of the regulatory measure, the EU has imposed limits on the use of P in the domestic cleaning products. However, such regulation does not apply to industrial products (Wentworth, 2014).

ii. Securing supply by enhancing P recycling, recovery and re-use

Though livestock manure is still extensively used around the world as fertilisers, it is unevenly distributed and remains concentrated in certain regions. The livestock production regions have excess of P while crop-growing districts run short for it. For sustainability, recycling of manure to maintain the balance across these regions is important but this demands technology, transportation and logistics. Technologies and facilities for manure dissemination exist but are used to only a limited extent, perhaps due to associated cost and inefficiencies.

P-rich bioresources such as composts, anaerobic digestates, municipal biosolids and biochars, are also used as the fertilisers. In addition, P is extracted from waste streams from swine and dairy farms, abattoirs, vegetable processing plants and other industrial by-products. The negative constraints on widespread use of such bioresources mainly include biogeochemical risks, energy efficiency, production cost and transportability. Interestingly, human excreta make up about ~14% of the globally lost phosphorus. If optimally recovered, it could satisfy up to 22% of the current global demand for P (Cordell & White, 2013). With population growth and the dietary shift, P concentrations in this bioresource could be even greater in future. However, the primary focus of the current sanitation systems is to remove waste while nutrient recycling for agriculture is secondary and challenging because of their diluted levels in centralised water-based systems and associated energy cost for the recovery.

There are numerous solutions for recovering phosphorus from waste sources, ranging in scale from small, such as direct urine use and composting faeces and solid bio-waste, to large, such as recovery of struvite from Wastewater Treatment Plants (WWTPs) (SCU, 2013). Different local scale initiatives for P recovery and reuse have been implemented in various countries including Sweden, Denmark, India, Niger and South Africa. Struvite recovered from WWTPs produces high quality of P fertilisers which is being used in treatment plants of some major cities in North America and UK. Besides these, P is also recovered from sewage sludge ash for industrial use in the Netherlands (Cordell *et al.*, 2011). In some European countries, the governments are encouraging P recovery from waste water streams and recycling it to land. For example, ICL fertiliser in Amsterdam has made legal agreement with Dutch government to use 15% recycled P in manufacturing of fertilisers by 2015 and aim to use 100% by 2025 (Wentworth, 2014).

There is great potential for recovery of phosphorus from mining remnants and thereby extending the life expectancy of reserves. It is estimated that around 15-30% of P is lost, during mining, processing and handling (Cordell & White, 2013). Furthermore, there is an additional environmental risk form the

presence of heavy or radioactive metals, like cadmium, radium and thorium, in the mining-waste products. However, technologies are being developed to address these issues. Globally, there is a trend towards improving recovery rates in the mining of lower grade P rock, whose use is limited by the higher impurity content (Wentworth, 2014) . Also, there are increasing interests and investments in exploring new phosphate rock deposits, most notably in Saudi Arabia, Australia and seabed sediments off the coast of Namibia (SCU, 2013).

In summary, the cost of recycled fertiliser is considerably higher today than the cost of imported mineral phosphorus fertiliser. It is essential to develop and implement technologies for safe and efficient extraction of P, along with identifying the trade-offs in using alternative bioresources. In the future, continued technological development and higher prices of mineral phosphorus are likely to result in the replacement of imported fertiliser with P.

II. Dynamics of phosphorus in soil

In nature, elemental phosphorus (P) always exists in combination with oxygen, mainly as "phosphate" anions. Depending on the source, phosphate in soil can be classed as in organic compounds (Po) and inorganic salts (Pi). Unlike other macronutrients, the concentration of soluble phosphate, mostly Pi, in the soil solution is often low, owing to its complex physico-chemistry, making it a major limiting factor for plant growth (Schachtman et al., 1998, Lambers and Plaxton, 2015). The dynamics of soil phosphate and its availability to plants, broadly depend upon: its forms and soil distribution, soil properties (physical, chemical and biological), environmental conditions, biotic content and agricultural practices.

Forms and distribution

Phosphorus is most abundant in the upper layers of soil, particularly in the top 15 cm and greatest in the top 5cm of soil (Jobbágy & Jackson, 2001). Distribution to any further depth in soil is either through addition of fertilisers or lysis of crop-root residues (Larsen, 1967). To some extent, its mobility and bioavailability depend upon its form. Soil phosphate can be broadly characterised as organic (Po) and inorganic (Pi), depending on their source. They differ in their behaviour and fate in soil (Shen et al., 2011) but are equally adept at supplying phosphate ions. Plants prefer Pi, but Po accounts for >50% of the total soil phosphate (Schachtman et al., 1998; Nash et al., 2014), acts as an important reservoir, and certain forms can be directly taken up by plants.

Being trivalent, phosphate is able to react with mono-, di- and trivalent cations. As a result, particles having a high anion exchange capacity (for example Al/Fe oxides and clays) will form strong bonds with phosphate producing stable compounds that are often insoluble and immobile. In contrast, anions, such as silicates, sulphates, and carbonates, compete with phosphate for sorption sites, increasing phosphate availability in the soil solution. This is known as mineralization. The presence of phosphate binding and competing particles varies with the soil type and its reaction with phosphate also depends on various other factors (Shen et al., 2011).

Under agronomic setup, soil phosphorus is "conceptually" characterised to exist in four different pools on the basis of their accessibility to plants (Johnston & Steen, 2000; Syers et al., 2008). The first phosphorus pool is the soil solution from where it is immediately take up plants. In the second pool, phosphorus is weakly bound to soil particles and can be readily transferred into soil solution during P limitation. Third pool consists of more strongly absorbed and less readily extractable phosphorus that can eventually become available to the plant, while the phosphorus in fourth pool has extremely low extractability, probably spanning over a period of many years. The inter-exchangeability of phosphorus

between different pools mainly relates to the differences in the bonding energy for the phosphate across the various sites within the soil matrix. Often, the bound phosphorus eventually tends to form less readily soluble compounds with strong bonding energies (Packer, 1998). This lost or trapped phosphate offers environmental risk in the form of eutrophication (Johnston & Steen, 2000).

Soil properties

Mineralization and immobilisation of phosphorus occurs simultaneously in the soil and the net process is determined by soil carbon : phosphate ratio (Marschner, 2008). Besides these, the release of Pi from Po is also affected by organic matter content, soil mineralogy, soil moisture, temperature and pH (Shen et al., 2011). Additionally, Pi loss is also influenced by the competition from Po for the sorption sites (Berg & Joern, 2006).

Compared to coarse-textured podsolized soils, fine-textured gleyed soil tends to release more phosphate through water extraction and allow a higher rate of diffusion. Soil aeration and compaction have opposite effects on phosphate diffusion. Compact soil has diminished pore spaces and a decreased thickness of water films on soil particles through which phosphate diffuses. This also limits oxygen in the soil, which restricts Pi absorption by roots. In contrast, well aerated soil enables roots to respire and produce the energy needed to exude metabolites that promote phosphate mineralisation. Soil pH is an important factor affecting phosphate speciation and availability. The maximum solubility and corresponding availability of Pi in most soil occur in pH range 6 – 7 (Schachtman et al., 1998).

Environmental effects

Soil phosphate exists as primary and secondary minerals. Primary phosphate minerals are very stable and release phosphate in the available form by quite slowly weathering process in order to meet with the crop demand. On contrast, secondary phosphate minerals have variable dissolution rates depending on the particle size and soil pH. Weathering of the parent material contributes to the pool of available soil phosphate (Cross & Schlesinger, 1995).

Low temperature and moisture reduces availability of soil phosphorus and its uptake by plants. Seasonally snow-covered temperate soils are subjected to freezing and thawing cycles (FTCs), which stimulate soil mineralisation and lysis of residual plant cells thereby elevating available Pi and Po during early spring. With the given projection of increase in air temperature in coming decades, cold and temperate regions may encounter an increase in number of FTCs due to reduced snow coverage. This could result in either an increase in or loss by runoff of available phosphate in early spring (Ziadi et al., 2013).

Drying and rewetting cycles in soil follow rainfall of particularly high intensity and short duration. This rapidly alters soil water-content and in turn concentration of available phosphate. During rewetting, the levels rise as a result of disintegration of occluded soil organic matter and increased microbial activity. The latter is thought to utilise soluble organic P compounds which then get released during drying phase. Unfortunately, there is little information in the literature on the long-term impacts of climate change on this process (Ziadi et al., 2013).

Biotic effects

Phosphate availability is increased by mycorrhizal symbiosis, microflora, plant root architecture and exudation. Mycorrhizal hyphae can be very long and carry Pi from large distances away to the plant, in exchange for photosynthate. Soil biota slowly converts Po into Pi by mineralisation, thereby

increasing phosphate levels, and it has been observed that the Po fraction drops during crop growth. The release of exudates from root and biota reduce the pH with the effect of solubilising Pi, and the root structure provides a network for rain water to get to where it will most benefit phosphate solubilisation. Altogether, these biological controls significantly alter soil physico-chemical properties and phosphate availability (Bünemann et al., 2011; Simpson et al., 2011; Hinsinger et al., 2015).

Effect of agricultural practices

From an agronomics perspective, soil phosphate exists in four or five different pools on the basis of their accessibility by plants (Johnston & Steen, 2000; Syers et al., 2008). The first pool is the soil solution from where it is immediately taken up plants. In the second pool, phosphate is weakly bound to soil particles and can be readily transferred into soil solution during phosphate limitation. Third pool consists of more strongly absorbed and less readily extractable phosphate that can eventually become available to the plant, while that in the fourth pool has extremely low extractability, probably spanning over a period of many years.

The first and second pools contribute most toward available phosphate. Its exchangeability between different pools mainly relates to the differences in its bonding energy across the various locations within the soil matrix, which tends to form less readily soluble compounds as stated above (Packer, 1998). This lost or trapped phosphate offers additional environmental risk in the form of eutrophication (Johnston & Steen, 2000). It is, therefore, important to determine soil phosphate levels so that the optimum level of fertiliser can be applied to the soil. The level achieving 90-95% of maximum crop yield is referred to as "critical soil-P level", and several tests have been developed for this purpose (Wolf & Baker, 1985; Kruse et al., 2015). The interpretations of different tests might vary and do not always correlate. In long-term field experiments on four different soil types, the critical soil-P ranged from 10 to 28 mg/kg for the optimal yields of wheat, maize and rice (Bai et al., 2013).

The type of mineral phosphate fertiliser significantly affects the soil physico-chemical properties (Shen et al., 2011), thus, it is essential to identify an appropriate sort for the soil properties in the field. In acidic soil, the application of powdered phosphate rock showed relatively efficient crop growth, though, even after applying bulk quantities of mineral fertilisers, crops barely capture 15-20% of the total phosphate applied (Schachtman et al., 1998). Other ions are also applied in fertilisers. These might not only affect the soil chemistry, as noted above, but also the ability of crops themselves to take up phosphate. Thus, sufficient nitrate is needed for efficient phosphate acquisition (Panigrahy et al., 2009). Agricultural practices such as crop rotation, tillage and fertilisation also have an impact on phosphate availability. Crop species and varieties vary in their root systems and soil organic-matter deposition, affect both their uptake efficiency and the available-phosphate level. To avoid stratification in the topsoil, ploughing and harrowing (i.e. tillage) allow the mixing of phosphate throughout the ploughing layer (Ziadi et al., 2013).

III. Dynamics of phosphorus in rhizosphere and plants

The volume of soil around living roots that is directly influenced by various root activities, including growth, uptake, respiration and rhizo-deposition, is referred to as the rhizosphere (Hinsinger *et al.*, 2005; McNear Jr., 2013; York et al. 2016). Depending on the considered activity, this root-soil interface ranges from the sub-μm to supra-cm scale (Hinsinger *et al.*, 2009). It forms the primary site for nutrient availability (Syers *et al.*, 2008). To meet with plant demand, soluble phosphate in the soil solution should be replaced 20 to 50 times per day by delivering more from bulk soil into the rhizosphere. Owing to its

low availability and slow diffusion (10^{-12} to 10^{-15} m^2s^{-1}) in soil, high phosphate uptake rates create a depletion zone (~ 0.2 -1 mm) and concentration gradient away from the root surface in the rhizosphere (Hinsinger et al., 2015). The above mentioned biotic effects particularly come into play here.

The primary sites in plants for the uptake of phosphate from the soil solution are the root tips, epidermal cells (especially those with root hairs) and cortical cells (Raghothama & Karthikeyan, 2005). As the concentration of phosphate ions in soil solution and apoplasm (<10 μM) is far less than that in cytosol (5-17 mM), epidermal and cortical cells acquire phosphate against an electrochemical potential gradient using an active co-transport process. It will also move towards the stele via plasmodesmata to supply phosphate to the rest of the plant through xylem vessels (Smith, 2002; Smith *et al.*, 2003). The concentration of Pi in xylem ranges from 1 mM to 7 mM in phosphate deficient and sufficient plants, respectively (Mimura *et al.*, 1996). The gradient of hydrostatic pressure and water potential in the xylem drives the transport of Pi across the plant in response to physiological demand (Smith *et al.*, 2003). Phosphate can be transferred between phloem and xylem via the few intermediary cambial cells separating them, so that it can reach the tissues where it is most needed.

In general, root-acquired Pi is transported to the younger leaves through the xylem, while phosphorus from older leaves is returned to the phloem as a mixture of Pi and Po (Schachtman *et al.*, 1998).When shoot-to-root translocation exceeds demand in the root, a considerable transfer of Pi back to the shoot via the xylem is suspected to occur in roots (Jeschke *et al.*, 1997). Inside the cell, Pi participates in various processes with destinations being dependent on the tissue type and metabolic requirement. This include production of nucleic acids, phospholipids and metabolites such as phosphor-esters and phosphorylated proteins (Veneklaas *et al.*, 2012). For proper functioning of plant processes, it is essential to maintain cytosolic Pi within appropriate levels. Within cells, this is achieved by tightly regulating the phosphate concentration in each organelle.

On entering a cell, Pi traverses the membrane of different organelles in exchange for other solutes or protons. Vacuoles serve as the storehouse, accounting for 85-95% of total cellular phosphorus (Pratt *et al.*, 2009). They act as a source or sink to maintain cytosolic phosphate levels. Vacuolar Pi uptake occurs via both ATP- and pyrophosphate-responsive proton pumps (Rausch & Bucher, 2002). Pi flux across the membrane of other organelles such as mitochondria, plastids and golgi, also involves an array of phosphate transporters that also affect Pi allocation across the plant (Shen *et al.*, 2011, Zhu *et al.*, 2012).

In the life cycle of a plant, most phosphate is absorbed during the vegetative phase of growth. During the reproductive stages, approximately 65-85% of this phosphate accumulates as K, Mg, Fe and Zn salts of phytic acid in the developing seed (Raboy, 2009). The enzyme phytase releases the phosphate from the grain during germination. Hence, seed phytate is the major source of phosphorus to support seedling establishment, especially in P-deficient soil (Marschner, 1995).

References:

Ahemad M, Kibret M. 2014. Mechanisms and applications of plant growth promoting rhizobacteria: Current perspective. Journal of King Saud University - Science 26(1): 1-20.
Bai Z, Li H, Yang X, Zhou B, Shi X, Wang B, Li D, Shen J, Chen Q, Qin W, Oenema O, Zhang F. 2013. The critical soil P levels for crop yield, soil fertility and environmental safety in different soil types. Plant and Soil 372(1-2): 27-37.
Berg AS, Joern BC. 2006. Sorption dynamics of organic and inorganic phosphorus compounds in soil. Journal of Environmental Quality 35(5): 1855-1862.
Bünemann E, Prusisz B, Ehlers K 2011. Characterization of Phosphorus Forms in Soil Microorganisms. in Phosphorus in Action (eds Bünemann E, Oberson A, Frossard E) Springer Berlin Heidelberg, 37-57.
Cordell D, Drangert J-O, White S. 2009. The story of phosphorus: Global food security and food for thought. Global Environmental Change 19(2): 292-305.
Cordell D, Rosemarin A, Schröder JJ, Smit AL. 2011. Towards global phosphorus security: A systems framework for phosphorus recovery and reuse options. Chemosphere 84(6): 747-758.
Cordell D, White S. 2013. Sustainable phosphorus measures: strategies and technologies for achieving phosphorus security. Agronomy 3(1): 86-116.
Cross AF, Schlesinger WH. 1995. A literature review and evaluation of the. Hedley fractionation: Applications to the biogeochemical cycle of soil phosphorus in natural ecosystems. Geoderma 64(3–4): 197-214.
Forsberg CW, Phillips JP, Golovan SP, Fan MZ, Meidinger RG, Ajakaiye A, Hilborn D, Hacker RR. 2003. The Enviropig physiology, performance, and contribution to nutrient management advances in a regulated environment: The leading edge of change in the pork industry. Journal of Animal Science 81(14): E68-E77.
Geneviève SMaEMBaJJE. 2012. The role of diet in phosphorus demand. Environmental Research Letters 7(4): 044043.
Hinsinger P, Gobran GR, Gregory PJ, Wenzel WW. 2005. Rhizosphere geometry and heterogeneity arising from root-mediated physical and chemical processes. New Phytologist 168(2): 293-303.
Hinsinger P, Bengough AG, Vetterlein D, Young IM. 2009. Rhizosphere: biophysics, biogeochemistry and ecological relevance. Plant Soil 321:117–152
Hinsinger P, Herrmann L, Lesueur D, Robin A, Trap J, Waithaisong K, Plassard C 2015. Impact of roots, microorganisms and microfauna on the fate of soil phosphorus in the rhizosphere. Annual Plant Reviews Volume 48: John Wiley and Sons, Inc., 375-407.
Jeschke WD, Kirkby EA, Peuke AD, Pate JS, Hartung W. 1997. Effects of P deficiency on assimilation and transport of nitrate and phosphate in intact plants of castor bean (Ricinus communis L.). Journal of Experimental Botany 48(1): 75-91.
Jobbágy E, Jackson R. 2001. The distribution of soil nutrients with depth: Global patterns and the imprint of plants. Biogeochemistry 53(1): 51-77.
Johnston AE, Poulton PR, Fixen PE and Curtin D 2014 Phosphorus: its efficient use in agriculture. Adv. Agron. 123:177-228.
Johnston AE, Steen I 2000. Understanding phosphorus and its use in agriculture. . Brussels - Belgium: European Fertilizer Manufacturers Association.
Lambers H and Plaxton WC .2015. Phosphorus: back to the roots. In Annual Plant Reviews Volume 48: Phosphorus Metabolism in Plants (eds W. C. Plaxton and H. Lambers), John Wiley and Sons, Inc., Hoboken, NJ, USA. pp. 3-22

Larsen S 1967. Soil Phosphorus. In: Norman AG ed. Advances in Agronomy: Academic Press, 151-210.

Marschner H 1995. 8 - Functions of Mineral Nutrients: Macronutrients. In: Marschner H ed. Mineral Nutrition of Higher Plants (Second Edition). London: Academic Press, 229-312.

Marschner P 2008. The role of rhizosphere microorganisms in relation to P uptake by plants. in The ecophysiology of plant-phosphorus interactions (eds. White P and Hammond J) Springer Netherlands, 165-176.

McLaughlin M, McBeath T, Smernik R, Stacey S, Ajiboye B, Guppy C. 2011. The chemical nature of P accumulation in agricultural soils—implications for fertiliser management and design: an Australian perspective. Plant and Soil 349(1-2): 69-87.

McNear Jr. DH. 2013. The Rhizosphere - Roots, Soil and Everything In Between. Nature Education Knowledge 4(3):1.

Mimura T, Sakano K, Shimmen T. 1996. Studies on the distribution, re-translocation and homeostasis of inorganic phosphate in barley leaves. Plant, Cell and Environment 19(3): 311-320.

Nash DM, Haygarth PM, Turner BL, Condron LM, McDowell RW, Richardson AE, Watkins M, Heaven MW. 2014. Using organic phosphorus to sustain pasture productivity: A perspective. Geoderma 221–222: 11-19.

Packer JE. 1998. The Fate of phosphate fertiliser in soils: New Zealand Institute of Chemistry.

Panigrahy M, Rao DN, Sarla N. 2009. Molecular mechanisms in response to phosphate starvation in rice. Biotechnology Advances 27(4): 389-397.

Pratt J, Boisson AM, Gout E, Bligny R, Douce R, Aubert S. 2009. Phosphate (Pi) starvation effect on the cytosolic Pi concentration and Pi exchanges across the tonoplast in plant cells: an in vivo 31P-nuclear magnetic resonance study using methylphosphonate as a Pi analog. Plant Physiology 151(3): 1646-1657.

Raboy V. 2009. Approaches and challenges to engineering seed phytate and total phosphorus. Plant Science 177(4): 281-296.

Raghothama KG, Karthikeyan AS 2005. Phosphate acquisition. Plant and Soil 274: 37–49

Rausch C, Bucher M. 2002. Molecular mechanisms of phosphate transport in plants. Planta. 216(1): 23-37.

Richardson A, Lynch J, Ryan P, Delhaize E, Smith FA, Smith S, Harvey P, Ryan M, Veneklaas E, Lambers H, Oberson A, Culvenor R, Simpson R. 2011. Plant and microbial strategies to improve the phosphorus efficiency of agriculture. Plant and Soil 349(1-2): 121-156.

Schachtman DP, Reid RJ, Ayling SM. 1998. Phosphorus Uptake by Plants: From Soil to Cell. Plant Physiology 116(2): 447-453.

Schröder JJ, Cordell D, Smit AL, Rosemarin A 2010. Sustainable Use of Phosphorus, prepared for European Union tender project ENV.B.1/ETU/2009/0025. Report 357. Wageningen: Plant Research International, Wageningen University and Research Centre. 122.

SCU (Science Communication Unit) 2013. Science for Environment Policy In depth Report: Sustainable Phosphorus Use. Prepared for the European Commission DG Environment. Bristol, UK: University of the West of England.

Sharma SB, Sayyed RZ, Trivedi MH, Gobi TA. 2013. Phosphate solubilizing microbes: sustainable approach for managing phosphorus deficiency in agricultural soils. Springer plus 2: 587.

Shen J, Yuan L, Zhang J, Li H, Bai Z, Chen X, Zhang W, Zhang F. 2011. Phosphorus dynamics: from soil to plant. Plant Physiology 156(3): 997-1005.

Simpson R, Oberson A, Culvenor R, Ryan M, Veneklaas E, Lambers H, Lynch J, Ryan P, Delhaize E, Smith FA, Smith S, Harvey P, Richardson A. 2011. Strategies and agronomic interventions to improve the phosphorus-use efficiency of farming systems. Plant and Soil 349(1-2): 89-120.

Smith FW. 2002. The phosphate uptake mechanism. Plant and Soil 245(1): 105-114.
Smith FW, Mudge SR, Rae AL, Glassop D. 2003. Phosphate transport in plants. Plant and Soil 248(1): 71-83.
SPS Committee 2012. Blueprint for Global Phosphorus Security. 3rd Sustainable Phosphorus Summit. Sydney, Australia: University of Technology
Syers J, Johnston E, Curtin D 2008. Efficiency of soil and fertilizer phosphorus use: Reconciling changing concepts of soil phosphorus behaviour with agronomic information. FAO - fertilizer and plant nutrition bulletin. Rome, Italy: FAO.
Veneklaas EJ, Lambers H, Bragg J, Finnegan PM, Lovelock CE, Plaxton WC, Price CA, Scheible WR, Shane MW, White PJ, Raven JA. 2012. Opportunities for improving phosphorus-use efficiency in crop plants. New Phytologist 195(2): 306-320.
Wentworth J 2014. Phosphate Resources. POSTnote (477) The Parliamentary Office of Science and Technology. London. UK.
Withers PJ, Sylvester-Bradley R, Jones DL, Healey JR, Talboys PJ. 2014. Feed the crop not the soil: rethinking phosphorus management in the food chain. Environmental Science and Technology 48(12): 6523-6530.
Withers PJ, van Dijk KC, Neset TS, Nesme T, Oenema O, Rubæk GH, Schoumans OF, Smit B, Pellerin S. 2015. Stewardship to tackle global phosphorus inefficiency: The case of Europe. Ambio 44(2): 193-206.
Wolf AM, Baker DE. 1985. Comparisons of soil test phosphorus by Olsen, Bray P1, Mehlich I and Mehlich III methods. Communications in Soil Science and Plant Analysis 16(5): 467-484.
York LM, Carminati A, Mooney SJ, Ritz K, Bennett MJ. 2016. The holistic rhizosphere: integrating zones, processes, and semantics in the soil influenced by roots. Journal of Experimental Botany 67(12): 3629–3643
Zhu W, Miao Q, Sun D, Yang G, Wu C, Huang J, Zheng C. 2012. The mitochondrial phosphate transporters modulate plant responses to salt stress via affecting ATP and gibberellin metabolism in Arabidopsis thaliana. PLoS One 7(8): e43530.
Ziadi N, Whalen JK, Messiga AJ, Morel C. 2013. Assessment and modeling of soil available phosphorus in sustainable cropping systems. Advances in Agronomy 122: 85-126.
